# Far infrared light irradiation enhances Aβ clearance via increased exocytotic microglial ATP and ameliorates cognitive deficit in Alzheimer’s disease-like mice

**DOI:** 10.1186/s12974-022-02521-y

**Published:** 2022-06-14

**Authors:** Qingyong Li, Jun Peng, Yuelian Luo, Jiaxin Zhou, Tailin Li, Lin Cao, Shuling Peng, Zhiyi Zuo, Zhi Wang

**Affiliations:** 1 Department of Anesthesiology, Sun Yat-Sen Memorial Hospital, Sun Yat-Sen University, No. 107 YanJiang West Road, Guangzhou, 510289 Guangdong Province China; 2 Medical Research Center, Sun Yat-Sen Memorial Hospital, Sun Yat-Sen University, Guangzhou, 510120 China; 3 Department of Anesthesiology, University of Virginia, Charlottesville, VA 22901 USA; 4 Department of Anesthesiology, University of Virginia Health System, 1 Hospital Drive, PO Box 800710, Charlottesville, VA 22908-0710 USA

**Keywords:** Far infrared light, Alzheimer’s disease, Amyloid-β clearance, Microglial phagocytosis, Energy mechanism

## Abstract

**Background:**

Exposure to sunlight may decrease the risk of developing Alzheimer’s disease (AD), and visible and near infrared light have been proposed as a possible therapeutic strategy for AD. Here, we investigated the effects of the visible, near infrared and far infrared (FIR) light on the cognitive ability of AD mice, and found that FIR light also showed potential in the improvement of cognitive dysfunction in AD. However, the related mechanism remains to be elucidated.

**Methods:**

Morris water maze was used to evaluate the cognitive ability of APPswe/PSEN1dE9 double-transgenic AD mice after light treatment. Western blot was carried out to detect the expression of protein involved in synaptic function and amyloid-β (Aβ) production. The protein amount of interleukin (IL)-1β, IL-6, Aβ_1-40_ and Aβ_1-42_ were determined using enzyme-linked immunosorbent assay. The mRNA level of receptors was performed using real-time quantitative polymerase chain reaction. Immunostaining was performed to characterize the Aβ burden and microglial Aβ phagocytosis in the brain of AD mice. The Aβ phagocytosis of primary cultured microglia and BV2 were assessed by flow cytometry. The energy metabolism changes were evaluated using related assay kits, including adenosine triphosphate (ATP), lactate content, mitochondrial respiratory chain complex enzymatic activity and oxidized/reduced nicotinamide adenine dinucleotide assay kits.

**Results:**

Our results showed that FIR light reduced Aβ burden, a hallmark of AD neuropathology, alleviated neuroinflammation, restored the expression of the presynaptic protein synaptophysin, and ameliorated learning and memory impairment in the AD mice. FIR light enhanced mitochondrial oxidative phosphorylation pathway to increase ATP production. This increased intracellular ATP promoted the extracellular ATP release from microglia stimulated by Aβ, leading to the enhanced Aβ phagocytosis through phosphoinositide 3-kinase/mammalian target of rapamycin pathways for Aβ clearance.

**Conclusions:**

Our findings have uncovered a previously unappreciated function of FIR light in inducing microglial phagocytosis to clean Aβ, which may be the mechanisms for FIR light to improve cognitive dysfunction in AD mice. These results suggest that FIR light treatment is a potential therapeutic strategy for AD.

**Supplementary Information:**

The online version contains supplementary material available at 10.1186/s12974-022-02521-y.

## Introduction

Alzheimer’s disease (AD) is the main cause of dementia and the most common neurodegenerative disease in the elderly, and is characterized by the presence of amyloid plaques and neurofibrillary tangles and a progressive loss in memory and cognitive function [1–3]. To date, there is no disease process-modifying intervention for AD [4]. It is estimated that about 150 million people worldwide will live with dementia in 2050 [3], which will be an enormous burden on the family, caregivers and society. Therefore, the prevention and treatment of AD are urgently needed. One major theory is that the imbalance between amyloid-β (Aβ) production and clearance results in the accumulation and aggregation of Aβ, which lead to many pathological processes in AD pathology, including neuroinflammation, oxidative stress and synaptic deficit [5, 6]. Therefore, it has been a focus to develop drugs that can eliminate the effects of Aβ. In the past decades, a large number of Aβ-related medications, such as semagacestat, bapineuzumab and solanezumab, had been proceeded to clinical trials but most of them failed to show beneficial effects [7, 8]. Various explanations for these failures have been suggested, including side effect, neuroinflammation response and wrong medication doses [7, 8]. It should be emphasized that these failures do not imply the fallacy of Aβ hypothesis [9]. Most recently, the aducanumab, an immunoglobulin G (IgG) 1 antibody targeting Aβ aggregates, had been approved by the US Food and Drug Administration for AD drug [10]. However, side effect occurs in AD patients following aducanumab treatment [11, 12]. Therefore, more effective and safe drugs or other therapeutic strategies are urgently needed [13, 14].

The combination of genetic, environmental and lifestyle factors have been recognized to play an important role in AD progression [15]. Interestingly, it was reported that exposure to sunlight was beneficial with a decreased risk for dementia [16, 17]. Old people with non-melanoma skin cancer (NMSC) probably caused by overexposure to the sunlight had a markedly reduced risk of developing AD as compared to whom without NMSC [18, 19]. These reports suggest that sunlight may have a great potential in the treatment of AD. So far, pharmacological therapeutic strategies have not taken advantage of the brain’s endogenous Aβ clearance mechanisms [14]. As the resident immune cells in the brain, microglia are able to carry out Aβ clearance [20, 21]. However, the Aβ phagocytic capacity of microglia decreases during AD progression, resulting in Aβ accumulation and therefore contributing to neurodegeneration [22]. To date, very little is known on how to manipulate these cells for enhanced Aβ clearance due to the lack of noninvasive methods [14]. It is worthwhile mentioning that sunlight, especially the infrared light, has transcranial potential [23]. Based on the findings of its beneficial effects on AD, sunlight exposure probably has a protective effect on the brain cells, including microglia.

The studies on using visible and near infrared light have suggested that these non-pharmacological and noninvasive treatments have the potential to improve AD due to their ability to alleviate pathology and cognitive dysfunction in AD mice [23–26]. As we know, sunlight has three major components: ultraviolet light, visible light and infrared radiation [27], and these lights also have multiple sub-divisions, such as near infrared light, middle infrared light and far infrared light. What sub-division of sunlight is beneficial in ameliorating cognitive dysfunction in AD remains largely elusive. Thus, we decided to investigate the effects of the visible light (*λ* = 500 nm), near infrared light (*λ* = 800 nm) and far infrared light (*λ* = 3–25 µm) on the cognitive ability of AD mice, and found that far infrared light also showed potential in the improvement of cognitive dysfunction in AD.

Of note, far infrared light has been reported to exert beneficial biological effects on animal and human [28, 29], as well as on many types of cells, including vascular endothelium, nerve, neuroblastoma, renal tubular cells and β-cells [30–34], and has the ability to improve mitochondrial function [32–34]. Mitochondria play an important role in the energy generation, and microglial Aβ phagocytosis has a high energy demand [35]. Thus, we hypothesized that far infrared light had beneficial biological effects on microglia, including improved mitochondrial energy metabolism and enhanced Aβ phagocytosis. To test this hypothesis, the AD-related neuropathology and biological responses of microglia in AD mice as well as the effect of mitochondrial energy metabolism on microglial Aβ phagocytosis were investigated.

## Materials and methods

### Animals

The APP_swe_/PSEN1dE9 double-transgenic (amyloid precursor protein (APP)/presenilin 1 (PS1)) AD mice and their littermate wild-type (WT) mice were obtained from Nanjing Biomedical Research Institute of Nanjing University (Nanjing, China). These mice were housed in specific pathogen-free environment with a 12-h light/12-h dark cycle. Mouse was caged singly and allowed to have food and water ad libitum. All experimental animal procedures were approved by the Institutional Animal Care and Use Committee (Approval No.: IACUC-20180918-07) and the Laboratory Animal Ethics Committee of Jinan University.

### Light irradiation treatment

At the age of 8.5 months, APP/PS1 mice were randomly distributed to the following groups: sham treatment group, the visible light (*λ* = 500 nm) treatment group (VIS light-treated APP/PS1 mice), the near infrared light (*λ* = 800 nm) treatment group (NIR light-treated APP/PS1 mice), and the far infrared light (*λ* = 3 to 25 μm) treatment group (FIR light-treated APP/PS1 mice). The mice in the VIS and NIR light treatment groups were irradiated with customized light-emitting diode (LED) based illuminating lamps (SD, Xuzhou Aijia Electronic Technology Co., Ltd, China). The two lamps contained the LEDs with the specific wavelength of 500 ± 10 nm and 800 ± 10 nm, respectively. The mice in the FIR light treatment group were irradiated with the FIR light emitter (JF-802, Guangdong Junfeng BFS Technology Co., Ltd, China), a ceramic FIR light generator that can emit infrared light with the wavelength range from 3 to 25 μm. A wild-type (WT) mouse group was also included. Mice in all groups were allowed free feeding during light irradiation that was for 60 min at 0.13 mW/cm^2^ per day for 1.5 months.

### Morris water maze

After 5-week treatment with different lights (VIS, NIR and FIR), all mice were subjected to Morris water maze behavior test under specific pathogen-free environment. The Morris water maze apparatus consisted of a circular pool with a diameter of 120 cm and a height of 50 cm, and water was injected into the pool. The camera that connected to a computer was located just above the tank, which was used to record the movement of mice. An appropriate amount of non-toxic protein powder was added into water to facilitate this recording. The pool temperature was maintained at 20–22 °C, and the water surface was divided into four quadrants and twelve zones. The water maze test was divided into two parts: the first is the training trial section (navigation stage of water maze), and the second is the spatial probe test (space memory stage). In the training trial, the target platform (8 cm in diameter, 20 cm in height) was located in the center of zone 2, zone 6 and zone 10 in the second quadrant, and the water surface was 1 cm higher than the target platform, so that the mice could not see the platform. The experimental parameters were as follows: swimming time (60 s) and residence time on target platform (3 s). During the water maze training trials, mice were placed into the water in accordance with the east and west directions. After mouse facing the pool wall was placed into the water, the time required to find the target platform was recorded, namely escape latency. Mice were allowed to stand on the target platform for 10 s. However, if mice were unable to find the target platform within 60 s, they were manually guided to find the target platform, and allowed to stay on it for 15 s. Each training cycle was done for 4 times and for 7 continuous days. In the spatial probe test that was 24 h after the last training trial, the target platform was removed. Mice were placed into the water again and observed for 60 s. The number of entries into effective zones to find the target platform was recorded to reflect the spatial memory of mice.

### Immunostaining

After the behavioral tests, the brains of the mice were harvested and bisected longitudinally. The left hemisphere was frozen at − 80 °C for further biochemical study. The right hemisphere was soaked in 4% paraformaldehyde solution for 24 h. Subsequently, they were immersed in 10%, 20% and 30% sucrose solutions each for 24 h and then stored in Tissue-Tek OCT compound at − 20 °C. Coronal 30-μm-thick sections from the right hemisphere were cut by a cryostat. The sections were washed with phosphate buffered saline (PBS).

For immunohistochemistry, the activity of endoperoxidase was blocked by 3% H_2_O_2_. Antigen retrieval with citric acid buffer (pH 6.0) was performed at 95 °C for 10 min. After washed with PBS, sections were incubated with 0.3% Triton X-100 and 5% donkey serum for 60 min at room temperature. The sections were then incubated with mouse anti-Aβ antibody (1:10,000, Sig-39300, Biolegend) overnight at 4 °C. Sections were then washed with PBS containing 0.3% Triton X-100 and incubated with goat anti-mouse/rabbit IgG conjugated with horseradish peroxidase (Universal kit, PV-600, Zhongshan Jinqiao, Beijing, China) at room temperature for 30 min. After being washed with PBS, sections were incubated with hydrogen peroxide (DAB kit, Zhongshan Jinqiao) and co-stained with hematoxylin [E803FA0003, Sangon Biotech (Shanghai) Co., Ltd, China] at room temperature for 5 min. Images were captured using a Nikon microscope (Nikon Ni-U).

For immunofluorescent staining, after being blocked with 0.3% Triton X-100 and 5% donkey serum at room temperature for 60 min, sections were then incubated at 4 °C overnight with the following primary antibodies: mouse monoclonal anti-Aβ (6E10) antibody (1:10,000, Sig-39300, Biolegend), rabbit polyclonal anti-ionized calcium binding adapter molecule 1 (Iba1) (1:1000, 019–19741, Wako) or rat polyclonal anti-cluster of differentiation (CD) 68 (1:2000, ab53444, Abcam). After rinsed with PBS containing 0.3% Triton X-100, the sections were incubated at room temperature for 2 h with fluorescent secondary antibodies: donkey anti-mouse IgG antibody conjugated with Alexa Fluor 488 (1:200, A21202, Invitrogen), donkey anti-rat IgG antibody conjugated with Alexa Fluor 488 (1:200, A21208, Invitrogen), donkey anti-rabbit IgG antibody conjugated with Alexa Fluor 555 (1:200, A31572, Invitrogen) or donkey anti-mouse IgG antibody conjugated with Alexa Fluor 647 (1:200, A31571, Invitrogen). Afterwards, the sections were incubated with 4′,6-diamidino-2-phenylindole (DAPI, C0065, Solarbio) for 10 min to stain the nuclei. Images were acquired using a ZEISS microscope (ZEISS Imager A2) or ZEISS confocal microscope (ZEISS LSM 800 with airyscan).

### Enzyme-linked immunosorbent assay

The protein amount of interleukin (IL)-1β, IL-6, Aβ_1-40_ and Aβ_1-42_ in the cerebral cortex were determined by using enzyme-linked immunosorbent assay kits (catalog No. E-EL-M0037c for IL-1β, catalog No. E-EL-M0044c for IL-6, catalog No. E-EL-H0542c for Aβ_1-40_, catalog No. E-EL-H0543c for Aβ_1-42_, Elabscience) according to the manufacturer’s instruction. Briefly, small pieces of the cerebral cortex of mice were collected. The tissue was then homogenized in PBS on ice with protease inhibitor cocktail for general use (P1005, Beyotime). Subsequently, the homogenates were centrifuged at 5000*g* for 8 min at 4 °C. The amount of IL-1β, IL-6, Aβ_1-40_ and Aβ_1-42_ in the supernatant was then determined. For the Aβ_1-40_ and Aβ_1-42_, after the PBS soluble fraction in the supernatant were collected, the pellets were further resuspended with guanidine hydrochloride (5 M) to extract the PBS-insoluble Aβ fraction as previously reported [36]. The final level of IL-1β, IL-6, Aβ_1-40_ and Aβ_1-42_ was then normalized to its protein content determined by a BCA protein assay kit (P0010, Beyotime).

### Western blot

The level of synaptic proteins from the hippocampus and key molecules involved in Aβ production from cerebral cortex were determined by using Western blot as previously described with slight modifications [37, 38]. The tissues were homogenized in the radio immunoprecipitation assay lysis buffer (CW23335, CWBIOTECH) by using 1-mL insulin syringe on ice with protease inhibitor cocktail for general use. Homogenates were centrifuged at 12,000*g* at 4 °C for 15 min. The supernatant was used for Western blot. The protein content of each sample was firstly determined, and twenty microgram protein per lane was electrophoresed on a 10% polyacrylamide gel and then transferred to a polyvinylidene fluoride membrane. The membrane was blocked by 5% nonfat milk at room temperature for 60 min and then incubated at 4 °C overnight with the following primary antibodies: mouse monoclonal anti-postsynaptic density protein-95 (PSD-95) antibody (1:1000, 75-028, NeuroMab), mouse monoclonal anti-synaptophysin (1:1000, MABN1193, EMD Millipore), rabbit polyclonal anti-APP (1:1000, AF6219, Beyotime), mouse monoclonal anti-Aβ (6E10) antibody that also recognized soluble APPα (sAPPα) (1:10,000, Sig-39300, Biolegend), rabbit polyclonal anti-nicastrin (1:1000, 14071-1-AP, Proteintech), rabbit polyclonal anti-beta-site-APP cleaving enzyme 1 (BACE1) (1:1000, AF6273, Beyotime), rabbit polyclonal anti-PS1 (1:1000, 16163-1-AP, Proteintech), or rabbit monoclonal anti-β-actin (1:1000, 8457 s, Cell Signaling Technology). After being washed with Tris-buffered saline Tween (Catalog No: T1081, Solarbio), the membranes were then incubated with corresponding secondary antibodies at room temperature for 2 h. The signal of protein band was visualized using NcmECL Ultra (Catalog No: P10200, Ncmbiotech). The protein band intensity of these proteins was normalized to the corresponding band intensity of β-actin from the same sample.

### Aβ plaque-associated microglial analysis

The quantification of Aβ plaque-associated microglia was similar to the previously reported method with minor modification [39]. Within 20 μm range from the edge of an Aβ plaque, the number of Iba1 staining positive cells was manually counted, which were performed by a person who was blind to group assignments. At least 100 plaques in the cerebral cortex region and 40 plaques in the hippocampus per group were quantified. For further analysis, plaques were divided into several groups according to their sizes (< 300, 300 to 600, 600 to 1200 and > 1200 µm^2^). The percentage of microglial CD68^+^ area was expressed as the co-staining area of Iba1^+^ and CD68^+^ divided by the total Iba1^+^ area. To further analyze the proximal interaction of microglia with Aβ plaque, the percentage of the co-staining area of 6E10^+^ and Iba1^+^ in total 6E10^+^ area was calculated. The co-staining area of 6E10^+^, CD68^+^ and Iba1^+^ divided by the total 6E10^+^ area was calculated to analyze the proximal interaction of CD68^+^ cells with Aβ plaque.

### Primary microglial culture

Primary microglial cultures were performed as previously described with slight modifications [40, 41]. Briefly, C57BL/6 mouse at postnatal 0 to 3 days was quickly immersed in 75% ethanol for disinfection process, and then the entire brain tissue was carefully removed with a sterile spatula. The olfactory bulbs, cerebellum and hind brain were dissected away, and the meninges were also removed. Subsequently, the cortical tissues were harvested, transferred to a 35-mm petri dish containing 2 mL Hanks' Balanced Salt Solution and minced into approximately 1 × 1 mm pieces by using sterile surgical scissors. The minced tissues were mechanically dissociated into suspension with a 1 mL pipette, and then filtered into a 50-mL conical tube by using a 40-μm mesh filter. The filtrated cell suspension was then transferred into a poly-l-lysine-coated T75 flask and cultured with Dulbecco’s modified Eagle’s medium (C1995500BT, Gibco) supplemented with 10% fetal bovine serum (SFBE, Natocor). After 3 days, medium was changed to that composed of 25 ng/mL granulocyte–macrophage colony-stimulating factor (415-ML-010, R&D system) and 10% fetal bovine serum. After being cultured for 12–14 days, microglial cells on top of the mixed glial cell layer were harvested by shaking the culture flask and were used for further experiment when their purity evaluated by Iba1 staining was over 95% (Additional file 1: Fig. S1A and S1B).

### Phagocytosis assay

The microglial phagocytosis of Aβ was analyzed similarly to a previously reported method with slight modifications [42]. Briefly, fluorescein amidite-labeled Aβ_1-42_ (FAM-Aβ_1-42_) (AS-23525, Anaspec) was first aggregated at 37 °C for 24 h with agitation. Microglia were seeded in the 6-well plates at a density of 1 × 10^5^ cells/cm^2^ (primary cultured microglia) or 5 × 10^4^ cells/cm^2^ (BV2 cells) and then cultured in medium containing 25 mM or 0.1 mM glucose overnight. In some experiments, primary cultured microglia were pre-incubated with 2-deoxy-D-glucose (2-DG) (5 mM, 2 h), antimycin A (0.5 μM, 2 h), suramin (100 μM, 30 min), apyrase (2 U/mL, 2 h), wortmannin (500 nM, 30 min) or rapamycin (100 nM, 30 min). After the cells were pretreated with FIR light (the same FIR light emitter used in the animal irradiation experiments mentioned above) at 0.13 mW/cm^2^ for 1 h, the aggregated FAM-Aβ_1-42_ was added into the culture medium with a final concentration of 0.8 μg/mL. The primary cultured microglia were continuously treated with FIR light at 0.13 mW/cm^2^ for an additional 3 h (irradiation of 1 h and then free-irradiation of 10 min, three cycles). As a negative control, the phagocytosis inhibitor cytochalasin D (10 µM, 11330, Cayman) was added for 30 min before the addition of FAM-Aβ_1-42._ Then FAM-Aβ_1-42_-containing cultured medium was removed and cultures were washed three times with PBS. Subsequently, the fluorescent images were randomly captured in five fields for each well by using an OLYMPUS microscope (OLYMPUS, IX71). For fluorescent intensity detection by flow cytometry, primary cultured microglia were digested with 0.25% trypsin-ethylenediaminetetraacetic acid (25200056, Gibco), centrifuged at 500*g* for 5 min at 4 °C and washed with PBS for three times. For the microscopic images, the green fluorescent intensity of internalized FAM-Aβ_1-42_ in each well, which was captured under a green fluorescent protein channel, was quantified based on the mean of fluorescent intensity from five random fields by using ImageJ 1.52n. The results of five images were averaged to reflect the phagocytosis activity of the cells in the well. In the case of detection by flow cytometry, the green fluorescent intensity of internalized FAM-Aβ_1-42_ from 10,000 cells in each sample was recorded under a fluorescein isothiocyanate (FITC) channel and then was expressed as the FITC mean for each sample. These measurements were performed by a person who was blind to group assignments. Finally, the green fluorescent intensity of treated samples was normalized to the mean fluorescent intensity of the control group without FIR light treatment.

### Intracellular and extracellular adenosine triphosphate measurements

Primary cultured microglia (3 × 10^5^) or mouse microglial cells line BV2 (1.5 × 10^5^) suspended in 1 mL medium containing 25 mM or 0.1 mM glucose were added to the 12-well plate overnight. In some experiments, the cells were pre-incubated with 2-DG (5 mM, 2 h), antimycin A (0.5 μM, 2 h), or apyrase (2 U/mL, 2 h). The medium was replaced with that containing vehicle or Aβ_1-42_ (0.1642 μM). Then the cell cultures were irradiated with FIR light at 0.13 mW/cm^2^ for 1 h or 3 h (irradiation of 1 h and then free-irradiation of 10 min, three cycles). For the intracellular adenosine triphosphate (ATP) measurements, the medium was removed and then the cells were washed with PBS. 100 uL ATP lysis solution (S0026-4, Beyotime) was added to the cells for 15 min under shaking condition. The lysis solution was collected and centrifuged at 12,000*g* for 5 min at 4 °C, and then the supernatants were collected for ATP measurement by using an ATP assay kit (S0026, Beyotime). For the extracellular ATP measurements, the medium was collected and centrifuged at 500*g* for 5 min at 4 °C. The supernatants were collected for ATP measurement by using an enhanced ATP assay kit (S0027, Beyotime). The final level of intracellular and extracellular ATP was then normalized to their cellular protein content.

### Lactate dehydrogenase cytotoxicity assay

Lactate dehydrogenase (LDH) activity was determined by using LDH cytotoxicity assay kit (C0017, Beyotime) as done previously with slight modification [37]. Briefly, primary cultured microglia suspended in 0.5 mL were added to the 24-well plate overnight. The culture medium was replaced with that containing vehicle or Aβ_1-42_ (0.1642 μM), followed by treatment with FIR light at 0.13 mW/cm^2^ for 1 h. The medium collected from 24-well plates at the end of experiments were centrifuged at 500*g* for 5 min at 4 °C and 120 μL of cell-free supernatant was transferred to 96-well plate. After removal of the medium from 24-well plates, 1% Triton X-100 lysing solution was added to the remaining cells and incubating for 15 min under shaking condition. Then the 1% Triton X-100 lysing solution was centrifuged at 12,000*g* for 5 min at 4 °C and 120 μL supernatant was transferred to 96-well plate for LDH activity measurement. The final percentage of LDH released to the medium was calculated as follows: spontaneously released LDH in the medium/(spontaneously released LDH in the medium + intracellular LDH released by 1% Triton X-100) × 100.

### Real-time quantitative polymerase chain reaction analysis

The total RNA was isolated from primary cultured microglia treated as indicated in the text using an RNA-Quick Purification kit (RN001, Yishan). Total RNA at 500 ng was reversely transcribed into the cDNA using Hifair® III 1st Strand cDNA Synthesis SuperMix for quantitative polymerase chain reaction (qPCR) (gDNA digester plus) kit (11141ES60, Yeasen). Real-time qPCR was performed using a Hieff UNICON® qPCR SYBR Green Master Mix kit (11198ES08, Yeasen) and achieved on 480 LightCycler (Roche). The primer sequences are listed in Additional file 1: Table S1. The mRNA level of these genes was normalized to that of β-actin from the same sample.

### Measurement of lactate

The intracellular lactate content was measured using a lactate colorimetric assay kit (E-BC-K044-M, Elabscience) according to the manufacturer’s instruction. Briefly, 5 × 10^5^ primary cultured microglia treated with or without FIR light at 0.13 mW/cm^2^ for 1 h were collected. The medium was removed and the cells were washed with PBS. PBS at 200 μL was added and cells were homogenized on ice. After that, the homogenates were centrifuged at 10,000*g* at 4 °C for 10 min. The supernatant was used for the lactate measurement. The lactate content was finally normalized to its protein content.

### Enzymatic activity of mitochondrial respiratory chain complex

Microglial mitochondrial respiratory chain complex I (BC0515, Solarbio), II (BC3235, Solarbio), III (BC3245, Solarbio), IV (BC0945, Solarbio) and ATP synthase (BC1445, Solarbio) activities were performed using corresponding assay kits according to the manufacturer’s instruction with slight adjustments. Briefly, at least 1.2 × 10^7^ BV2 cells treated with or without FIR light at 0.13 mW/cm^2^ for 1 h were collected. After the medium were removed, 1 mL extract buffer was added to each sample, and homogenized on ice with a homogenizer. Subsequently, the homogenates were centrifuged at 500*g* for 5 min at 4 °C. The supernatants were transferred to another centrifugal tube, and centrifuged at 11,000*g* for 10 min at 4 °C. After the supernatants were removed, 400 μL extract buffer were added and these homogenates were then subjected to the ultrasonic lysis (power 52 W, ultrasonic 5 s, interval 10 s, for 15 times). The supernatant is used for mitochondrial respiratory chain complex activity determination. Finally, the enzymatic activity of these complexes was normalized to its protein content.

### NAD^+^ level and ratio of NAD^+^/NADH

The oxidized nicotinamide adenine dinucleotide (NAD^+^) content and the ratio of NAD^+^/reduced NAD (NADH) were measured using NAD^+^/NADH assay kit with WST-8 (S0175, Beyotime) according to the manufacturer’s instruction. Briefly, 1 × 10^6^ BV2 cells treated with or without FIR light at 0.13 mW/cm^2^ for 1 h were collected. The medium was removed and the cells were washed with PBS. NAD^+^/NADH extract buffer at 200 μL was added and gently pipetting to promote cell lysis. After that, the cell lysate was collected and centrifuged at 12,000*g* at 4 °C for 5 min, and the supernatant was used for the NAD^+^ content and the ratio of NAD^+^/NADH determination. The NAD^+^ content was finally normalized to its protein content.

### Statistical analysis

All data were expressed as means ± standard error of the mean (SEM). The statistical analysis of results was performed by using GraphPad Prism version 8.0. The significant difference was assessed by unpaired t test, one-way or two-way analysis of variance (ANOVA) followed by Turkey’s or Dunnett multiple comparisons test. The data of training sessions in the Morris water maze test, Aβ phagocytosis of microglia or intracellular ATP level of microglia treated with FIR light and cytochalasin D, or cultured in the medium containing antimycin A and different concentrations of glucose were analyzed by two-way repeated measures ANOVA. A *p* value < 0.05 was considered statistically significantly different.

## Results

### FIR light ameliorated cognitive dysfunction of AD mice

**Fig. 1:**
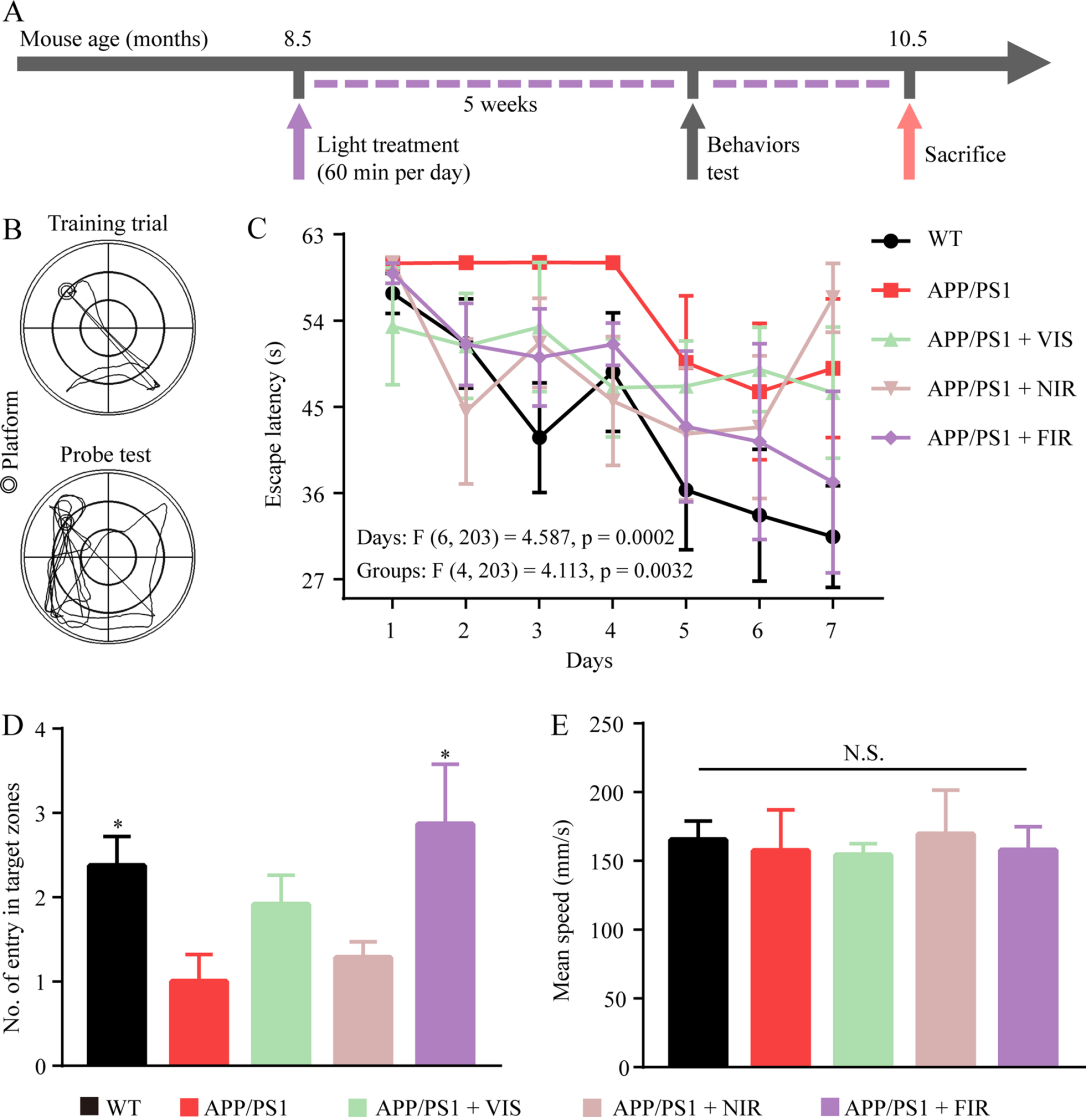
FIR light ameliorated learning and memory deficit of AD mice. **A** Timeline of treatment with visible light (VIS), near infrared light (NIR) and far infrared light (FIR) on APP/PS1 mice. **B** Representative tracking routes of mice in training trials and spatial probe test. **C** The escape latency of mice to find the platform during training trials. **D** The number of entries in the zones around platform. **E** The mean swimming speed of mice. Data were means ± SEM, *n* = 5–8, *p < 0.05 compared to APP/PS1 mice without light treatment. N.S., not significant

The time-course of the in vivo experiments is shown in Fig. 1A. The Morris water maze test consisted of two stages: training trials and spatial probe test (Fig. 1B). WT mice took a shorter time than APP/PS1 mice to find the platform in the training trials of the test (Fig. 1C). This impaired learning in the APP/PS1 mice was improved by the treatment of various wavelengths of light [F (4, 203) = 4.113, *p* = 0.0032]. Interestingly, mice in different groups during training stage had an increase in escape latency on one day compared to that on the prior day. For instance, VIS and NIR light-treated APP/PS1 mice had an increased escape latency on day 3 compared with that on day 2, and FIR light-treated APP/PS1 mice had an increase in escape latency on day 4 compared with that on day 3. This phenomenon was found in other studies [43–45]. Although this phenomenon can result in statistical difference in the performance of mice between different treatment groups, there was no significant difference (*p* = 0.1837) in the escape latency on day 7 between NIR and FIR light-treated mice. Compared to APP/PS1 mice without treatments, FIR light-treated but not VIS light- or NIR light-treated APP/PS1 mice had more entries into the zones around the platform in the spatial probe test (Fig. 1D). Notably, there was no difference in swimming speed among WT mice and APP/PS1 mice treated with or without various lights (Fig. 1E). These results suggested that FIR light improved the spatial memory of AD mice and later studies were thereby focused on the effects of FIR light on AD mouse brain.

### FIR light reduced Aβ burden in the brain of AD mice

**Fig. 2:**
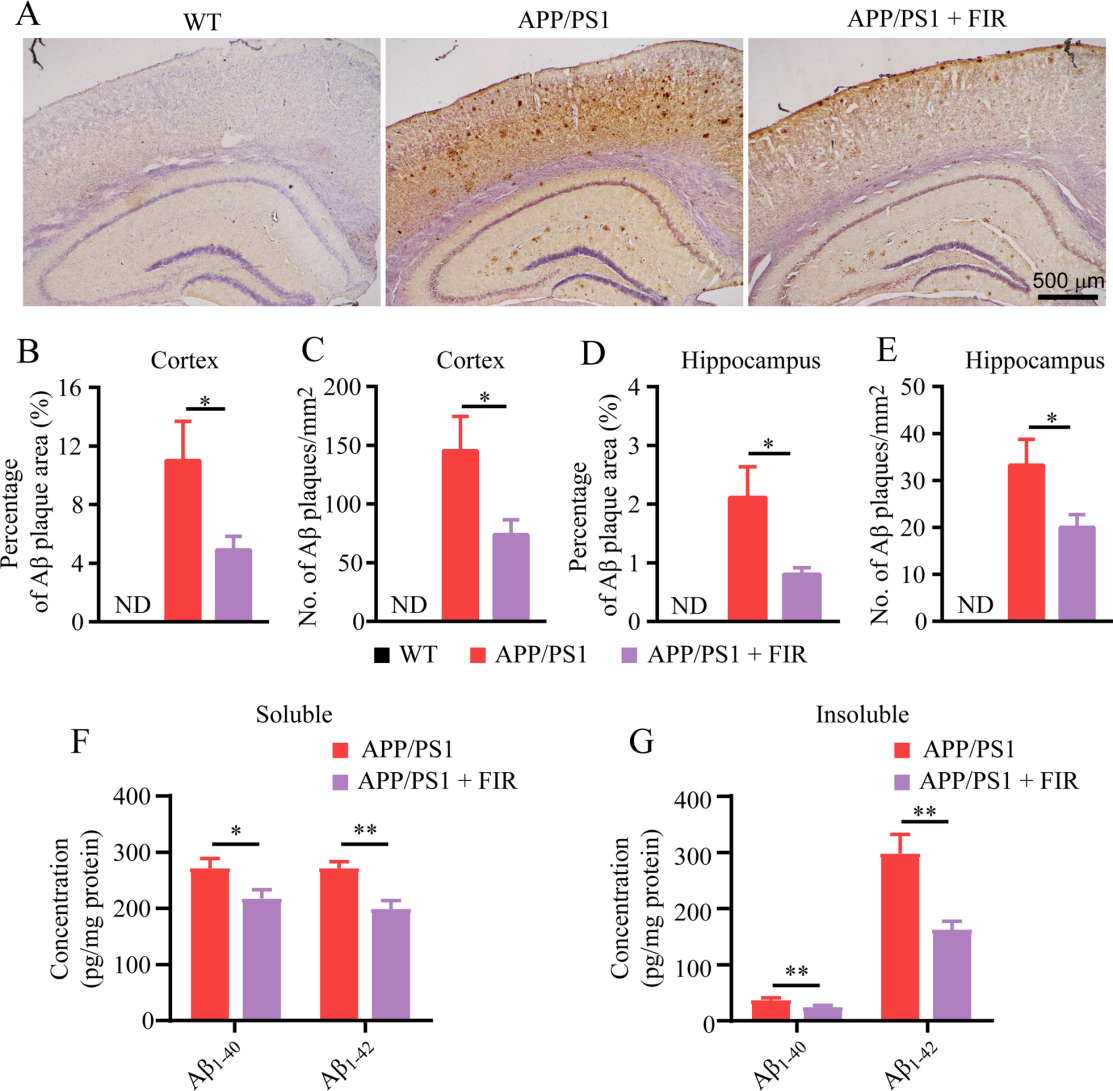
FIR light treatment ameliorated Aβ deposition in the cortex and hippocampus. **A** Representative images of Aβ (6E10) staining in the cerebral cortex and hippocampus from WT, APP/PS1 and FIR light-treated APP/PS1 mice. **B** Percentage of Aβ plaque area in the cerebral cortex (*n* = 4). **C** Aβ plaque density in the cerebral cortex (*n* = 4). **D** Percentage of Aβ plaque area in the hippocampus (*n* = 4). **E** Aβ plaque density in the hippocampus (*n* = 4). **F** The levels of Aβ_1-40_ and Aβ_1-42_ in the PBS fractions from the cerebral cortex (*n* = 5). **G** The levels of Aβ_1-40_ and Aβ_1-42_ in the guanidine hydrochloride fractions from the cerebral cortex (*n* = 5). Data were means ± SEM, *p < 0.05, **p < 0.01. ND: not detected

Aβ pathology is one of the most distinctive hallmarks of AD, and these Aβ accumulation and deposition in the brain would result in a series of subsequent pathological events, including neuroinflammation, impaired neuronal function and cognitive dysfunction for AD progression [6, 46]. We next determined whether FIR had an beneficial effect on the alleviation of Aβ burden in the brain of AD mice. Compared to WT mouse, APP/PS1 mouse exhibits abundant Aβ plaque in both cerebral cortex and hippocampus (Fig. 2A). A significant reduction of Aβ plaque in these brain areas of the FIR light-treated APP/PS1-mice was found compared to the APP/PS1 mice (Fig. 2B–E). The levels of Aβ_1-40_ and Aβ_1-42_ in the PBS and guanidine hydrochloride fractions from cerebral cortex were markedly lower upon the FIR light treatment (Fig. 2F and G). These results indicate that FIR light significantly reduces Aβ level, resulting in decreased Aβ load in the brain of APP/PS1 mice.

We next determined whether FIR light alleviated neuroinflammation in the AD mice. As shown in Additional file 1: Fig. S2A and S2B, the proinflammatory factors including IL-1β and IL-6 were significantly higher in the APP/PS1 mice compared to WT mice. However, they were markedly reduced in the FIR light-treated APP/PS1 mice. In addition, we examined the expression of pre- and post-synaptic proteins including synaptophysin and PSD-95 due to their pivotal role in regulating synaptic plasticity, which closely correlates with neuronal function and cognitive functions [47, 48]. Western blot results showed that the level of PSD-95 did not exhibit significant changes in APP/PS1 mice compared with WT mice (Additional file 1: Figs. S3A and S3B). However, the level of synaptophysin was significantly decreased in the APP/PS1 mice compared to the WT mice, but this decrease was attenuated in the APP/PS1 mice treated with FIR light (Additional file 1: Figs. S3A and S3C). These results suggest that FIR light effectively reduces Aβ burden and neuroinflammation and restore the expression of synaptophysin in the brain of AD mice.

### FIR light did not influence the Aβ production but enhanced microglial Aβ phagocytosis

**Fig. 3:**
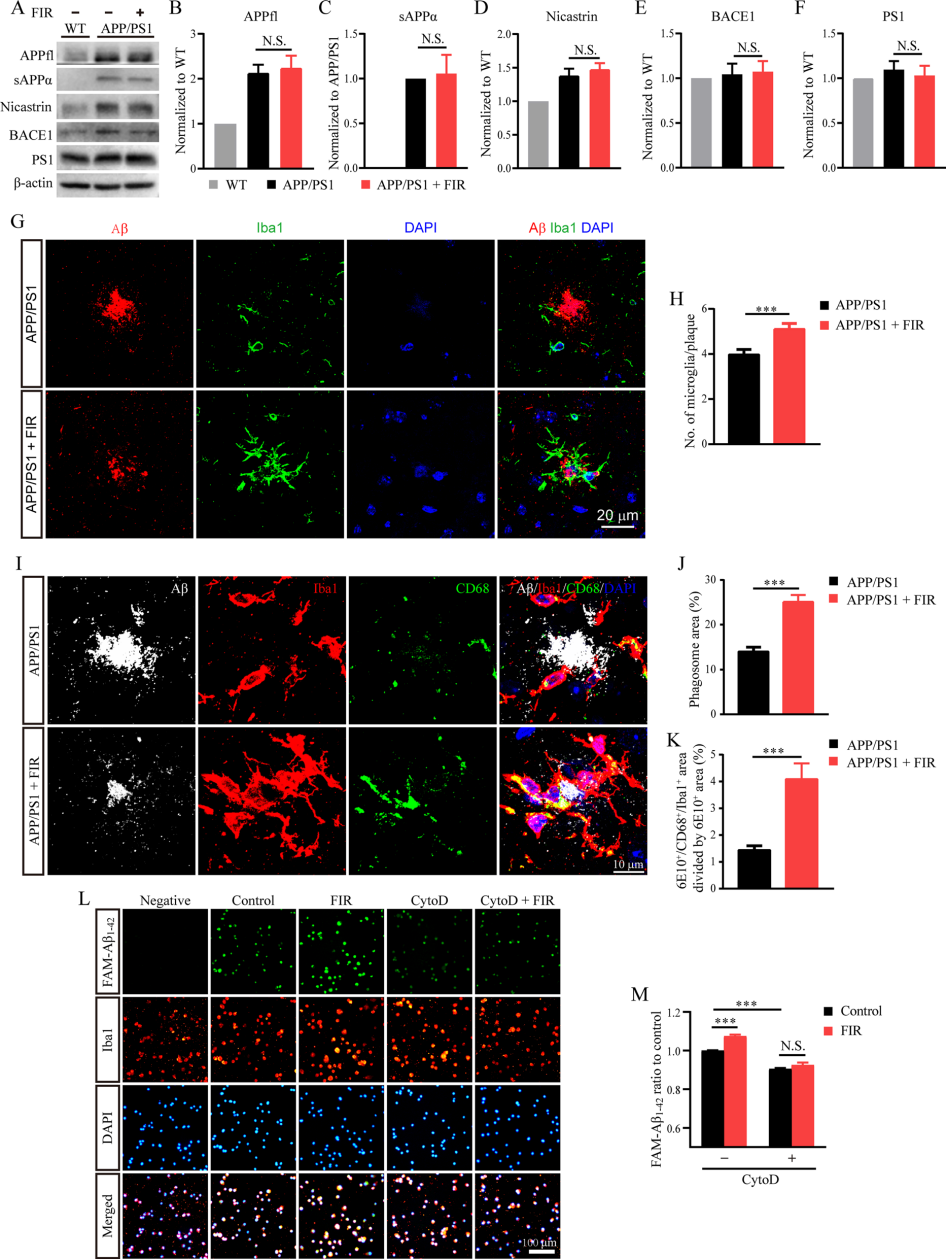
FIR light did not influence the Aβ production but enhanced microglial Aβ phagocytosis. **A** The Western blot analysis of key molecules involved in the process of Aβ production. Expression level of **B** APPfl, **C** sAPPα, **D** Nicastrin, **E** BACE1 and **F** PS1 (*n* = 5 per group). **G** Representative images of Aβ (6E10, red), microglia (Iba1, green) and nuclei (DAPI, blue) co-staining from APP/PS1 treated with or without FIR light. **H** Quantification of microglial cells within 20 μm from the Aβ plaque boundary (*n* = 102 to 103 plaques per group) in the cerebral cortex. **I** Representative images of Aβ (6E10, white), microglia (Iba1, red), phagosome (CD68, green) and nuclei (DAPI, blue) co-staining in the cerebral cortex of APP/PS1 mice treated with or without FIR light. **J** Quantification of the percentage of phagosome area in Iba1 positive area (*n* = 49–52 per group). **K** Quantification of the percentage of 6E10^+^/CD68^+^/Iba1^+^ co-staining area normalized to the total 6E10^+^ area (*n* = 49–52 per group). **L** Representative images of the uptake of FAM-Aβ_1-42_. **M** Image quantification of the corresponding fluorescent intensity of FAM-Aβ_1-42_ engulfed by microglia (*n* = 6 per group). Data were means ± SEM, ***p < 0.001. N.S., not significant. CytoD: cytochalasin D

The Aβ accumulation and aggregation are mainly resulted from the imbalance of Aβ production and clearance [5]. Given the finding that FIR light was able to reduce the Aβ burden in the brain of AD mice, we asked whether FIR light could decrease Aβ production or increase Aβ clearance. We first examined a series of key molecules that are implied in the production of Aβ. The Western blot results showed that there was no significant change in the protein expression of amyloid precursor protein [APP full length (APPfl)], and APP processing secretases, including BACE1, and γ-secretase complex composed of nicastrin and PS1, between control and FIR light-treated APP/PS1 mice (Fig. 3A–F), suggesting that FIR light did not influence the process of Aβ production. Accordingly, we speculated that FIR light reduced Aβ burden through the clearance pathway. As the resident immune cells in the brain of AD, microglia are responsible for the clearance of Aβ [49]. In the present study, numerous microglia were recruited to the Aβ plaques (Fig. 3G). In the cortical region, FIR light enhanced the recruitment of microglia surrounding the Aβ plaques (Fig. 3H and Additional file 1: Fig. S4A). The number of microglia surrounding the Aβ plaques was increased with an increased size of Aβ plaque and FIR light increased the number of microglia no matter which size of Aβ plaque was considered (Additional file 1: Fig. S4B). Similar results were observed in the hippocampus (Additional file 1: Fig. S4C–S4E).

To investigate whether the microglia surrounding the plaques could engulf Aβ to achieve Aβ clearance, co-immunostaining of Iba1, CD68 and Aβ was carried out, and the CD68^+^ microglial phagosomes and internalized Aβ were quantified. The results showed that the microglia around the Aβ plaque expressed the phagocytic marker CD68 (Fig. 3I). Compared to the microglia in the control APP/PS1 mice, the microglia in the FIR light-treated APP/PS1 mice had a significant increase in CD68 expression (Fig. 3J). In addition, the interaction between microglia and Aβ was enhanced due to the increase in their contact area and phagocytic area in the FIR light-treated APP/PS1 mice (Additional file 1: Figs. S4F and 3 K), suggesting that FIR light enhanced microglial Aβ phagocytosis and clearance.

To further confirm whether FIR light could enhance microglial Aβ phagocytosis, mouse primary cultured microglia were used to assess FAM-Aβ_1-42_ uptake. The co-immunostaining results showed that microglia could engulf FAM-Aβ_1-42_ (Fig. 3L), and FIR light markedly enhanced the microglial capacity to engulf FAM-Aβ_1-42_ (Fig. 3M), which was accompanied by an increased mRNA level of putative microglial phagocytic receptors, including triggering receptor expressed on myeloid cells 2 (Trem2), Toll-like receptor (TLR) 2, TLR4, CD14, integrin alpha M (Itgam), scavenger receptor class B type 1 (Scarb1) and Mer receptor tyrosine kinase (MerTK) (Additional file 1: Fig. S4G). However, this enhanced phagocytic effect was almost abolished in the present of the phagocytic inhibitor cytochalasin D (Fig. 3M), which was verified by measurements with the flow cytometry (Additional file 1: Figs. S4H and S4I). These results were in agreement with the findings that FIR light was able to significantly enhance Aβ phagocytosis of microglia in the APP/PS1 mice.

### FIR light promoted ATP production involved in the enhanced microglial Aβ phagocytosis

**Fig. 4:**
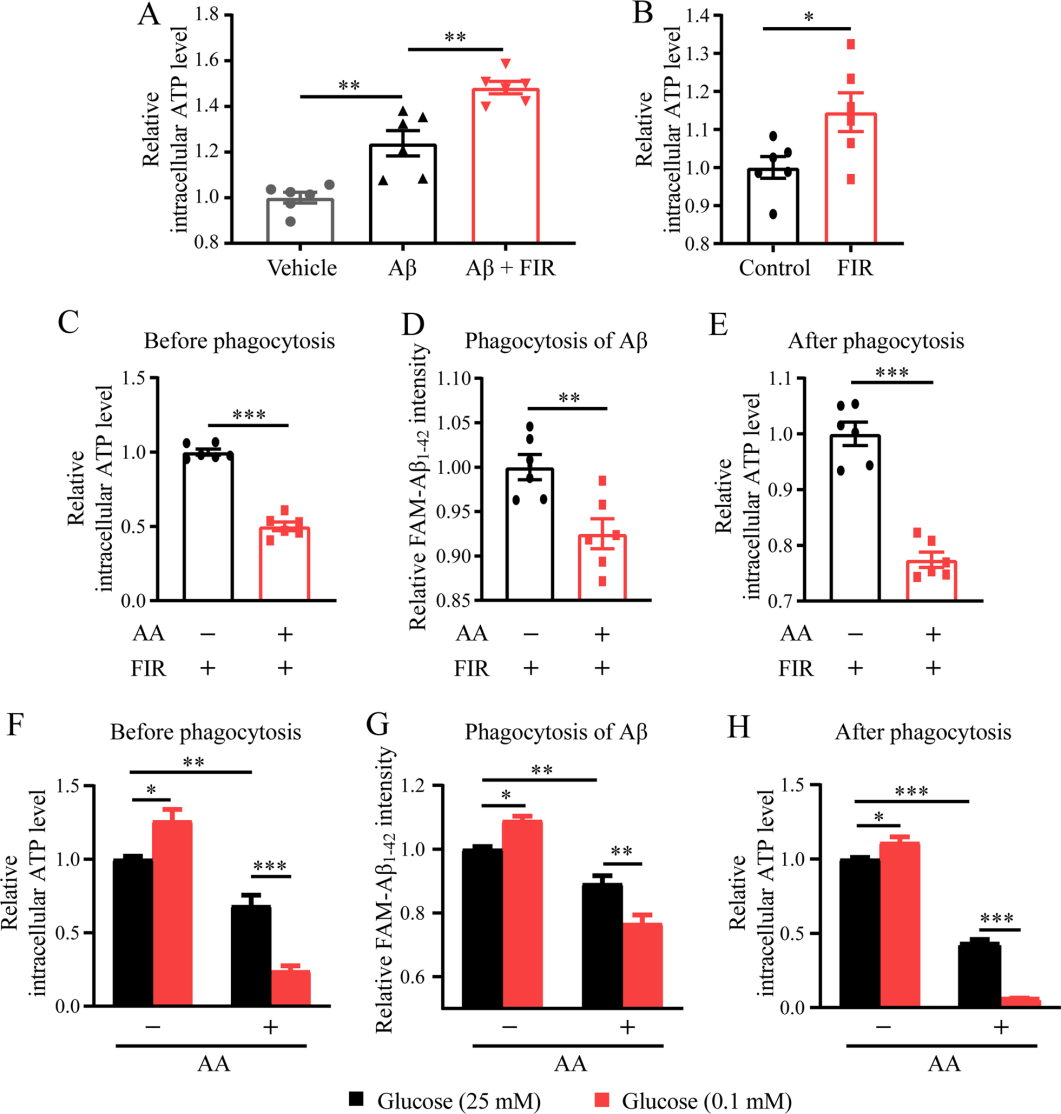
Increased ATP production was involved in the FIR light-enhanced microglial Aβ phagocytosis. **A** Intracellular ATP of microglia under different conditions. **B** Intracellular ATP of microglia exposed to FIR light. Upon the FIR light treatment, **C** before Aβ phagocytosis, intracellular ATP of microglia treated with antimycin A or not, **D** Aβ phagocytosis of microglia treated with antimycin A or not, **E** after Aβ phagocytosis, intracellular ATP of microglia treated with antimycin A or not. Upon the treatment with antimycin A or not, **F** before Aβ phagocytosis, intracellular ATP of microglia cultured in medium containing glucose of 25 mM and 0.1 mM, respectively, **G** Aβ phagocytosis of microglia cultured in medium containing glucose of 25 mM and 0.1 mM, respectively, **H** after Aβ phagocytosis, intracellular ATP of microglia cultured in medium containing glucose of 25 mM and 0.1 mM, respectively. Data were means ± SEM, *n* = 6, *p < 0.05, **p < 0.01, ***p < 0.001. AA: antimycin A

Since microglial phagocytosis requires dynamic reorganization of the actin cytoskeleton, a substantial amount energy is necessary [50]. We observed that the decreased intracellular ATP level in the microglia cultured in glucose-free medium or pretreated with the glycolytic inhibitor 2-DG was accompanied by decreased microglial Aβ phagocytosis (Additional file 1: Fig. S5A–S5D), indicating that intracellular ATP may play a pivotal role in the microglial Aβ phagocytosis. Recent studies on other types of cells treated with FIR light show improvement in mitochondrial function [32–34], which can promote energy metabolism. Accordingly, we asked whether FIR light could promote microglial energy metabolism and consequently enhanced microglial Aβ phagocytosis. As shown in Fig. 4A, we observed that intracellular ATP was markedly increased in microglia upon Aβ stress. Intracellular ATP level in the Aβ-induced microglia was further elevated with FIR light treatment (Fig. 4A). We found that FIR light could increase the intracellular ATP even without Aβ stress (Fig. 4B).

Tricarboxylic acid (TCA) cycle coupling with mitochondrial oxidative phosphorylation (OXPHOS) was the major pathway to produce ATP. To further investigate the relationship between the increase in the intracellular ATP and the enhancement in the microglial Aβ phagocytosis after FIR light treatment, the mitochondrial OXPHOS inhibitor antimycin A was used to reduce ATP production (Fig. 4C). This reduced ATP production was accompanied by the compromised FIR light-enhanced microglial Aβ phagocytosis (Fig. 4D) and the decreased intracellular ATP even under Aβ stress (Fig. 4E). Since glucose concentration in cultured medium influences the ATP production in cells [51, 52], we tried to change intracellular ATP in the microglia by culturing them in medium containing different glucose concentrations to further investigate the relationship between intracellular ATP and microglial Aβ phagocytosis under the condition without FIR light treatment. The results showed that increased intracellular ATP in microglia cultured in the medium containing 0.1 mM glucose was accompanied by enhanced microglial Aβ phagocytosis, as compared to that of microglia cultured in the medium containing 25 mM glucose (Fig. 4F and G). Decreased intracellular ATP was found in microglia cultured in the medium containing antimycin A, which was associated with decreased microglial Aβ phagocytosis (Fig. 4F and G). This intracellular ATP change was not altered even under Aβ stress (Fig. 4H). Interestingly, upon the antimycin A pretreatment, the intracellular ATP was further decreased in the microglia cultured in the medium containing 0.1 mM than 25 mM glucose, which was associated with more compromised Aβ phagocytosis by microglia (Fig. 4F–H). These results clearly suggest the important role of intracellular ATP in the microglial Aβ phagocytosis. These findings also suggest that FIR light promotes ATP production and such an increase in ATP was involved in the enhanced Aβ phagocytosis of microglia treated with FIR light.

### FIR light enhanced microglial Aβ phagocytosis through increased ATP release due to increased ATP production

**Fig. 5:**
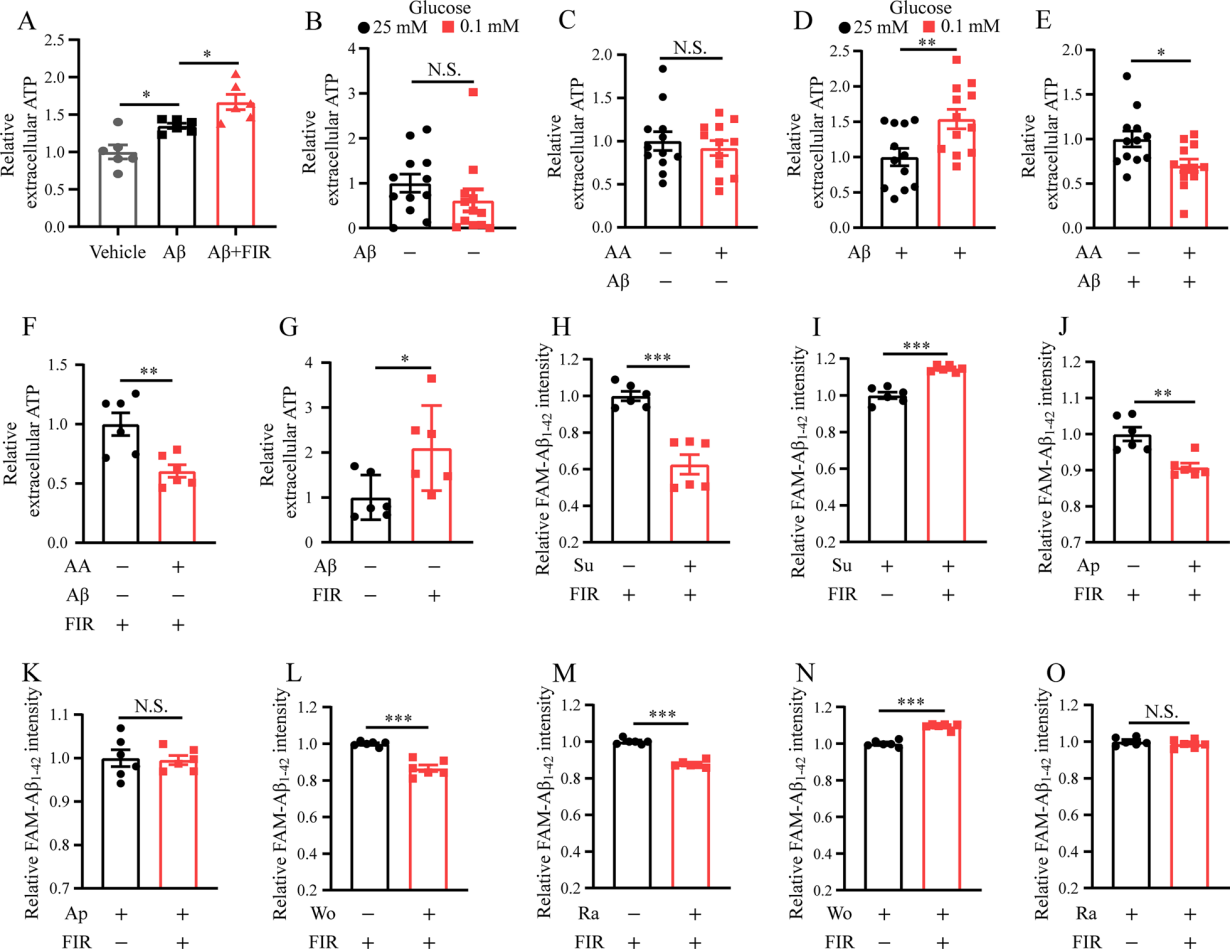
FIR light enhanced microglial Aβ phagocytosis through increased microglial extracellular ATP release. **A** Extracellular ATP of microglia under different stimulation conditions. Without Aβ stimulation, extracellular ATP of microglia cultured in **B** medium containing glucose of 25 mM and 0.1 mM, respectively (*n* = 12), or **C** medium containing glucose of 25 mM and additionally adding AA or not (*n* = 12). With Aβ stimulation, extracellular ATP of microglia cultured in **D** medium containing glucose of 25 mM and 0.1 mM, respectively (*n* = 12), or **E** medium containing glucose of 25 mM and additionally adding AA or not (*n* = 12). **F** Upon the FIR light treatment, extracellular ATP of microglia treated with antimycin A or not (*n* = 6). **G** Extracellular ATP of microglia treated with FIR light or not (*n* = 6). **H** Upon the FIR light treatment, Aβ phagocytosis of microglia pretreated with suramin or not (*n* = 6). **I** Upon the suramin pretreatment, Aβ phagocytosis of microglia treated with FIR light or not (*n* = 6). **J** Upon the FIR light treatment, Aβ phagocytosis of microglia pretreated with apyrase or not (*n* = 6). **K** Upon the apyrase pretreatment, Aβ phagocytosis of microglia treated with FIR light or not (*n* = 6). **L** Upon the FIR light treatment, Aβ phagocytosis of microglia pretreated with wortmannin or not (*n* = 6). **M** Upon the FIR light treatment, Aβ phagocytosis of microglia pretreated with rapamycin or not (*n* = 6). **N** Upon the wortmannin pretreatment, Aβ phagocytosis of microglia treated with FIR light or not (*n* = 6). **O** Upon the rapamycin pretreatment, Aβ phagocytosis of microglia treated with FIR light or not (*n* = 6). Data were means ± SEM, *p < 0.05, **p < 0.01, ***p < 0.001. N.S.: not significant. AA: antimycin A. Su: suramin. Ap: apyrase. Wo: wortmannin. Ra: rapamycin

Previous study has shown that microglia would release ATP responding to the Aβ stress [53]. ATP has been recently reported to modulate microglial Aβ phagocytosis [54]. In the present study, microglia showed increased extracellular ATP level when stimulated by Aβ (Fig. 5A), and this Aβ-induced increase was further elevated when microglia was exposed to the FIR light (Fig. 5A). To rule out the possibility that ATP release caused by Aβ or Aβ plus FIR light was due to microglial lysis, medium was analyzed for LDH release at the end of experiments. The results exhibited that Aβ or Aβ plus FIR light did not significantly cause microglial LDH release (Additional file 1: Fig. S6A), suggesting that this increased ATP release was not caused by microglial lysis.

We then asked whether increased ATP production would spontaneously promote extracellular ATP release. To answer this question, extracellular ATP level was analyzed after increasing and decreasing the ATP production of microglia induced by 0.1 mM glucose and antimycin A, respectively, as described above (Fig. 4F). The results exhibited that 0.1 mM glucose or antimycin A did not significantly change extracellular ATP level as compared to the level from microglia cultured in the medium containing 25 mM glucose and without antimycin A pretreatment, respectively (Fig. 5B and 5C). However, upon the treatment with Aβ, extracellular ATP levels were significantly increased and decreased, respectively (Fig. 5D and E). Moreover, this antimycin A-induced decrease in extracellular ATP level was unchanged even with the FIR light treatment (Additional file 1: Fig. S6B), suggesting that ATP production influences extracellular ATP release for the microglia stimulated by Aβ. Interestingly, microglia pretreated by antimycin A reduced extracellular ATP release when they were exposed to FIR light (Fig. 5F). Next, we asked whether FIR light could induce extracellular ATP release just like Aβ stress. The results exhibited that FIR light promoted microglial extracellular ATP release without cell lysis, which was verified by LDH assay (Fig. 5G and Additional file 1: Fig. S6C). Nevertheless, Aβ induced additional microglial ATP release upon the FIR light treatment (Additional file 1: Fig. S6D). Therefore, elevated ATP production of microglia treated with FIR light was beneficial to the extracellular ATP release of microglia stimulated by Aβ.

In addition, we observed that FIR light changed the mRNA levels of microglial ATP-related receptors, including P2X1, P2X5, P2X7, P2Y2, P2Y6, P2Y13, P2Y14 (Additional file 1: Fig. S6E and S6F), upon the Aβ stimulation, indicating that the increase in Aβ-induced extracellular ATP release of microglia treated with FIR light influences the ATP-related signal transduction. We blocked these ATP-related receptors by using suramin, a broad-spectrum antagonist of P2 purinoceptors [55], to investigate the role of extracellular ATP in the enhanced Aβ phagocytosis of microglia treated with FIR light. The results showed that suramin compromised the Aβ phagocytosis of microglia in the presence or absence of FIR light (Fig. 5H and Additional file 1: Fig. S6G), suggesting that extracellular ATP is involved in the FIR light-enhanced microglial Aβ phagocytosis. However, suramin did not stop the enhancement in Aβ phagocytosis of microglia treated with FIR light (Fig. 5I), which might be due to a limited ability of suramin to inhibit these ATP-related receptors. Thus, we next directly decreased the extracellular ATP level through the pretreatment with apyrase (Additional file 1: Fig. S6H to S6J), an enzyme that hydrolyzes extracellular ATP [56]. The results exhibited that addition of apyrase markedly decreased the Aβ phagocytosis of microglia treated with FIR light (Fig. 5J) or cultured in medium containing 25 mM or 0.1 mM glucose (Additional file 1: Figs. S6K and S6L). Notably, apyrase effectively abrogated the ability of FIR light to enhance microglial Aβ phagocytosis (Fig. 5K), including the microglia cultured in the medium containing 0.1 mM glucose (Additional file 1: Fig. S6M).

Additionally, it has been reported that phosphoinositide 3-kinase (PI3K) and mammalian target of rapamycin (mTOR) pathways can be activated by ATP and affect the endocytosis of microglia [54, 57]. To investigate whether PI3K and mTOR pathway was involved in the FIR light-induced enhanced microglial Aβ phagocytosis, their corresponding inhibitors wortmannin and rapamycin were used to block these pathways. As shown in Fig. 5L, M, Additional file 1: Fig. S6N and S6O, wortmannin and rapamycin decreased the Aβ phagocytosis of microglia treated with or without FIR light. Moreover, rapamycin but not wortmannin was capable of suppressing the enhancement in Aβ phagocytosis of microglia treated with FIR light (Fig. 5N and O), suggesting that PI3K and mTOR pathways are involved in the FIR light-enhanced microglial Aβ phagocytosis.

### FIR light promoted ATP production mainly through mitochondrial OXPHOS pathway

**Fig. 6:**
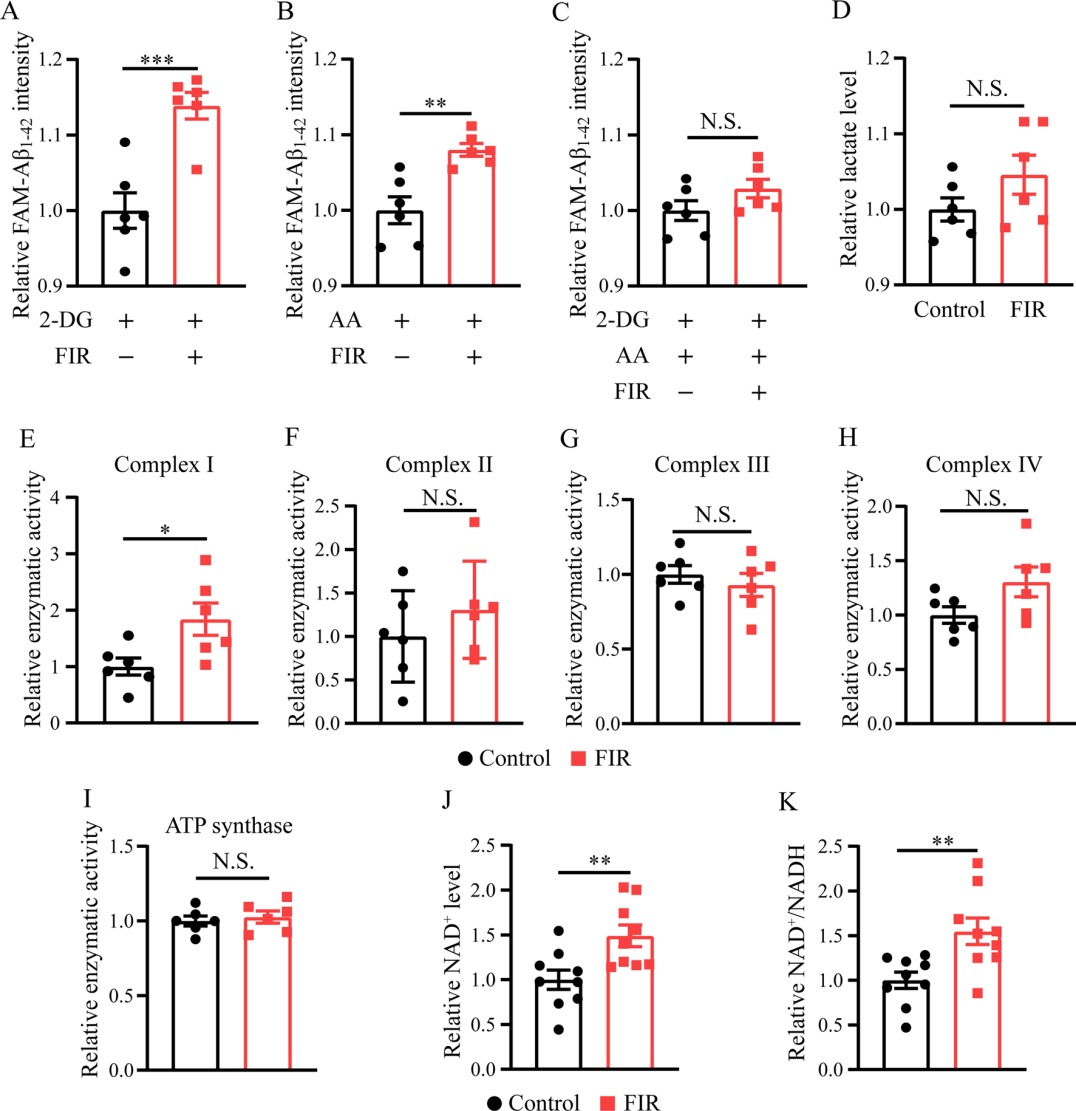
FIR light promoted microglial ATP production mainly through mitochondrial OXPHOS pathway. **A** Upon the 2-DG pretreatment, Aβ phagocytosis of microglia treated with FIR light or not (*n* = 6). **B** Upon the antimycin A pretreatment, Aβ phagocytosis of microglia treated with FIR light or not (*n* = 6). **C** Upon the 2-DG plus antimycin A pretreatment, Aβ phagocytosis of microglia treated with FIR light or not (*n* = 6). **D** The lactate level of microglia treated with FIR light or not (*n* = 6). The enzymatic activity of **E** complex I, **F** complex II, **G** complex III, **H** complex IV, and **I** ATP synthase in the mitochondrial respiratory chain in BV2 microglia (*n* = 6). **J** The NAD^+^ level and **K** the ratio of NAD^+^/NADH of BV2 microglia treated with FIR light or not (*n* = 9). Data were means ± SEM, *p < 0.05, **p < 0.01, ***p < 0.001. N.S.: not significant. AA: antimycin A

Given that increased ATP production was critical in the enhanced Aβ phagocytosis of microglia exposed to FIR light and that glycolysis and TCA cycle coupling with OXPHOS in the mitochondria are the two major pathways to produce ATP, the effects of FIR light on microglial energy metabolism pathway were investigated. 2-DG and antimycin A were used to block glycolysis and OXPHOS pathway, respectively, and then to monitor the Aβ phagocytosis of microglia treated with FIR light. The results exhibited that 2-DG or antimycin A did not suppress the enhancement in Aβ phagocytosis of microglia treated with FIR light, although both were able to decrease the microglial Aβ phagocytosis under the condition of FIR light treatment (Fig. 6A, B, Additional file 1: Fig. S7A and 4D). However, 2-DG plus antimycin A effectively abrogated the ability of FIR light to enhance microglial Aβ phagocytosis (Fig. 6C). Interestingly, compared with antimycin A pretreatment, FIR light had a greater positive effect on Aβ phagocytosis (increase of 13.9% and 8.0% in the microglia pretreated with 2-DG and antimycin A, respectively) in 2-DG-pretreated microglia (Fig. 6A and B), suggesting that OXPHOS pathway effectively affects the enhanced Aβ phagocytosis of microglia treated with FIR light. Thus, increased ATP production of microglia treated with FIR light might be mainly through the OXPHOS pathway.

We further examined the production of lactate and the enzymatic activity of the mitochondrial respiratory chain complexes (complex I, II, III, IV and ATP synthase), which are closely associated with the glycolysis and OXPHOS pathways, respectively. As shown in Fig. 6D, lactate level was not significantly increased in the microglia treated with FIR light. In the present study, microglial BV2 cell line was used to investigate the effect of FIR light on the enzyme activity of these mitochondrial complexes due to their ability to generate adequate cells for enzymatic activity assay. BV2 cells had a increased ATP production and enhanced Aβ phagocytosis when exposed to FIR light (Additional file 1: Figs. S7B to S7D), which was similar to that of mouse primary cultured microglia. The results showed that FIR light markedly improved the enzyme activity of complex I but had no significant effect on the enzyme activity of complex II, III, IV and ATP synthase (Fig. 6F–I). Since complex I in the mitochondria converts NADH to NAD^+^, we monitored the NAD^+^ level and the ratio of NAD^+^ to NADH. The results showed that the NAD^+^ level and the ratio of NAD^+^ to NADH were significantly increased in BV2 treated with FIR light (Fig. 6J and K), which was in agreement with the increased enzyme activity of the complex I in BV2 following FIR light treatment.

## Discussion

During the progression of AD, the most common symptom is the decline in cognitive function including poor memory and learning skill. Accordingly, improving cognitive dysfunction has been a goal of AD treatment. Interestingly, it has been reported that sunlight exposure could decrease the risk of developing AD [16–19]. Sunlight mainly contains three lights, namely ultraviolet light, visible light and infrared light, as well as their multiple sub-divisions, such as near infrared light and far infrared light [27, 29]. However, it is still largely unclear what sub-division of sunlight has beneficial in ameliorating cognitive dysfunction in AD. In the present study, VIS, NIR and FIR light were tested as a treatment strategy. APP/PS1 mice with different light treatments showed a trend of improvement in learning compared to those without light treatment during training stage. Notably, FIR light-treated APP/PS1 mice had better spatial memory than APP/PS1 mice in the probe test, suggesting that FIR light show potential in the improvement of cognitive dysfunction in AD mice.

In the past decade, the potential of using VIS and NIR light as an AD therapeutic intervention had been investigated [23–26, 58], especially the NIR light. However, few studies had focused on the therapeutic effect of FIR light on the AD. FIR light irradiation that can penetrate up to 1.5 in. beneath the skin had been reported to have many positive biological effects on animal and human, including improving blood circulation, ameliorating endothelial dysfunction, relieving fatigue and pain, lowering blood pressure, and promoting capillary dilation [28, 29]. In the present study, we compared for the first time the effects of VIS, NIR and FIR light on the cognitive function of the 8.5-month-old APP/PS1 mice. It is reported that APP/PS1 mouse shows increased Aβ plaque and cognitive deficits by the age of 4 months and 6 months, respectively [59, 60]. Therefore, 8.5-month-old APP/PS1 mice are on the progressive stage of disease. Our results showed that only FIR light significantly improved the spatial memory in APP/PS1 mice. Further studies also showed multiple beneficial effects of FIR light on the AD mice, such as reduced Aβ plaque burden, decreased neuroinflammation and restored the expression of the presynaptic protein synaptophysin, indicating that FIR light treatment may have a potential to be a therapeutic strategy for AD.

Previous studies suggest that proper neuronal function is the underlying mechanism for cognitive ability [61–63]. However, excessive Aβ in AD would induce the release of proinflammatory cytokines and then cause neuronal damage [64–66], leading to synaptic function impairment. Synaptic plasticity deficit with decreased expression of synaptic protein can compromise normal neuronal functions, resulting in cognitive dysfunction, which often occurs in the AD brain [47, 67]. Here, we found that APP/PS1 mice treated with FIR light had a higher expression of presynaptic protein synaptophysin than those without FIR light treatment. This result be may a subsequence of the decrease in Aβ and proinflammatory cytokines after FIR light treatment. Together, these findings suggest that FIR light treatment may normalize synaptic integrity and restore proper neuronal function, which may be a mechanism for the improved cognitive function of AD mice.

Given that excessive Aβ drives the neuropathogenic cascades of AD [64, 65], reducing excessive Aβ in the brain has been proposed to be a potential therapeutic strategy for AD. Previous studies showed that light treatment could reduce Aβ burden in the brain of AD mice [23–26]. However, the mechanisms underlying the positive effect of light treatment on reducing Aβ remain to be fully elucidated. In this study, we found that FIR light reduced Aβ burden without affecting the process of Aβ production. Since the accumulation of Aβ in brain of AD mice is considered to be the disequilibrium between Aβ production and clearance [6, 68], we speculated that FIR light might enhance the Aβ clearance pathway. As the resident immune cells in brain, microglia may be the first responder to clean Aβ through phagocytosis [49]. Nevertheless, the phagocytic ability of microglia decreases with aging, leading to the reduction of Aβ clearance during AD development [22]. One particular study showed that 40-Hz white-light flicker can transform microglia into an engulfing state and reduce Aβ in AD mice [14]. A recent study demonstrated that NIR light of 1070 nm was able to induce microglial responses with change in morphology and increased colocalization with Aβ, which was sufficient to decrease Aβ burden in AD mice [69]. In this study, we focused on investigating the involvement of microglia in the underlying mechanism for FIR light to reduce Aβ burden in the brain of AD mice. Similarly, our results showed that FIR light promoted the recruitment of microglia to the Aβ plaque with increased CD68^+^ phagosomes and enhanced microglial Aβ phagocytosis both in vivo and in vitro, which are beneficial to the Aβ clearance.

**Fig. 7:**
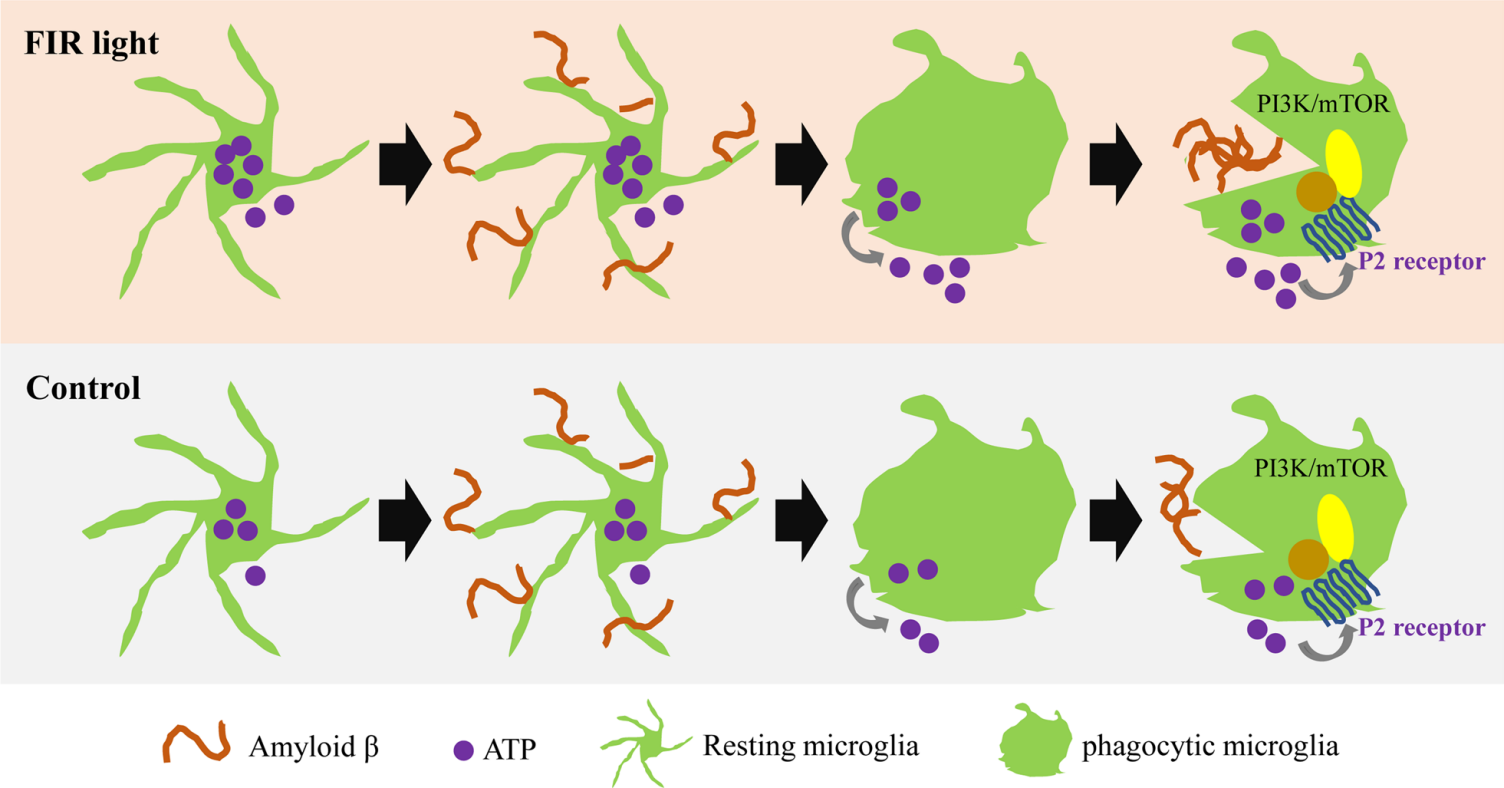
Schematic presentation showing the pathways that mediate the effects of FIR light on microglial Aβ clearance

It was previously speculated that a substantial amount of energy is necessary for microglial phagocytosis [35, 50, 70]. The ability of FIR light to improve cellular mitochondrial function had been reported [32–34], which would enhance cellular energy metabolism. However, the energy metabolism of microglia induced by FIR light as well as the relationship between the microglial Aβ phagocytosis and the energy metabolism of microglia treated with FIR light remain unclear. Here, our results showed for the first time that FIR light was able to promote the production of energy substance ATP in microglia and this increased intracellular ATP was involved in the enhanced microglial Aβ phagocytosis. This phenomenon of increased ATP production leading to enhanced Aβ phagocytosis also occurred in microglia treated with other strategies [9, 71]. It is worth noting that ATP is not only a universal intracellular energy substance but also acts as an important extracellular signaling molecule in cell processes [72, 73]. A previous study observed that microglia would release ATP when stimulated by Aβ [53], and microglial Aβ phagocytosis could be modulated by ATP [54]. In this study, we found that the increased intracellular ATP of microglia treated with FIR light promoted the extracellular ATP release from the microglia stimulated by Aβ, although the clear mechanism remained to be elucidated. Importantly, our further results demonstrated that this increased extracellular ATP mediated the enhancement of Aβ phagocytosis in the microglia treated with FIR light. Recent studies reported that PI3K and mTOR pathways were involved in the endocytosis of microglia induced by ATP [54, 57, 74]. Our current study found that PI3K and mTOR pathways were involved in the FIR light-enhanced microglial Aβ phagocytosis. Overall, increased ATP production of microglia treated with FIR light plays an indispensable role in the enhanced microglial Aβ phagocytosis (Fig. 7).

Our further study showed that FIR light had no significant effect on the microglial lactate production, which is associated with anaerobic glycolysis, but was able to improve enzymatic activity of the mitochondrial respiratory chain complex I and increase NAD^+^ level and the ratio of NAD^+^/NADH in the mitochondrial of microglia. These results are similar to the recent studies that FIR light could enhance mitochondrial function and increase the ratio of NAD^+^/NADH both in the NRK-52E cells and RIN-m5f cells [33, 34]. As we know, glycolysis and TCA cycle coupling with OXPHOS in the mitochondria are the two major pathways to produce ATP. Our results suggest that FIR light enhances the mitochondrial OXPHOS pathway, which is in agreement with the increased ATP production in the microglia treated with FIR light.

There are some limitations in this study. First, the design that wavelength of 500 nm, 800 nm and 3–25 μm represent as VIS, NIR and FIR light, respectively, is a type of simplification because the wavelengths of these lights cover an extremely broad range [27, 29]. Second, we compared the effects of VIS, NIR and FIR lights on the cognitive function of AD mice with the same power density and intervention time, and we found that FIR light showed a greater potential in the improvement of cognitive function of AD mice. However, the requirement of illumination parameters, such as fluence, power density and treatment timing, may be different for a specific light to be protective [75]. Detailed and systematic studies should be conducted to uncover the optimal light treatment in AD. Third, FIR light had the ability to increase microglial phagocytosis mediated by alteration in increased ATP production and ATP release. Additional experiments are needed to clarify the mechanism for these findings.

## Conclusions

In summary, our study revealed that FIR light at wavelengths of 3–25 µm could enhance mitochondrial OXPHOS pathway to increase ATP production. This increased intracellular ATP promoted the extracellular ATP release of microglia stimulated by Aβ, leading to the enhanced Aβ phagocytosis of microglia treated with FIR light through the PI3K/mTOR pathways, which was beneficial to the Aβ clearance. Thus, FIR light was able to reduce Aβ burden in the brain of AD mice, resulting in beneficial effects, including decreased neuroinflammatory cytokines and restored expression of presynaptic protein synaptophysin. As a result, FIR light ameliorated the learning and memory impairment of these AD mice, suggesting a therapeutic potential of FIR light for AD.

## Supplementary Information

**Additional file 1. **Additional Figures and Tables.

## Data Availability

The datasets generated during and/or analyzed during current study are available from the corresponding authors on a reasonable request.

## Abbreviations

AD: Alzheimer’s disease
Aβ: Amyloid-β
APP/PS1: Amyloid precursor protein (APP) and presenilin 1 (PS1)
WT: Wild-type
FIR: Far infrared
VIS: Visible light
NIR: Near infrared
LED: Light-emitting diode
qPCR: Quantitative polymerase chain reaction
NMSC: Non-melanoma skin cancer
PBS: Phosphate buffered saline
IgG: Immunoglobulin G
Iba1: Ionized calcium binding adapter molecule 1
DAPI: 4′,6-Diamidino-2-phenylindole
HBSS: Hanks' balanced salt solution
FAM-Aβ_1-42_: Fluorescein amidite-labeled Aβ_1-42_
IL-1β: Interleukin-1β
IL-6: Interleukin-6
PSD-95: Postsynaptic density protein 95
ATP: Adenosine triphosphate
2-DG: 2-Deoxy-d-glucose
LDH: Lactate dehydrogenase
BACE1: Beta-site APP-cleaving enzyme 1
Trem2: Triggering receptor expressed on myeloid cells 2
TLR2, TLR4: Toll-like receptor 2, 4
CD14, CD68: Cluster of differentiation 14, 68
Itgam: Integrin alpha M
Scarb1: Scavenger receptor class B type 1
MerTK: Mer receptor tyrosine kinase
OXPHOS: Oxidative phosphorylation
P2X1, P2X5, P2X7: ATP ligand-gated cation channel P2X receptor 1, 5, 7
P2Y2, P2Y6, P2Y13, P2Y14: ATP G protein-coupled P2Y receptor 2, 6, 13, 14
PI3K: Phosphoinositide 3-kinase
mTOR: Mammalian target of rapamycin
TCA: Tricarboxylic acid
NAD+, NADH: Oxidized, reduced nicotinamide adenine dinucleotide
