# Supplementary material for: Far infrared light irradiation enhances Aβ clearance via increased exocytotic microglial ATP and ameliorates cognitive deficit in Alzheimer’s disease-like mice
Source: J Neuroinflammation. 2022 Jun 14;19:145. doi: 10.1186/s12974-022-02521-y (PMC9195249; doi:10.1186/s12974-022-02521-y)
Supplement: Supplementary file 1 — Additional file 1. Additional Figures and Tables. [file 12974_2022_2521_MOESM1_ESM.docx]

Supplementary materials for:

**Far infrared light irradiation enhances Aβ clearance via increased exocytotic microglial ATP and ameliorates cognitive deficit in Alzheimer’s disease-like mice**

Qingyong Li^1,2,#^, Jun Peng^1,#^, Yuelian Luo^1^, Jiaxin Zhou^1^, Tailin Li^1^, Lin Cao^1^, Shuling Peng^1^, Zhiyi Zuo^3,^*, Zhi Wang^1,^*

^1^Department of Anesthesiology, Sun Yat-Sen Memorial Hospital, Sun Yat-Sen University, Guangzhou, 510289, China

^2^Medical Research Center, Sun Yat-Sen Memorial Hospital, Sun Yat-Sen University, Guangzhou, 510120, China

^3^Department of Anesthesiology, University of Virginia, Charlottesville, Virginia 22901, U.S.A.

^#^Equal contributors

*Correspondence: [zz3c@virginia.edu](mailto:zz3c@virginia.edu); [wangzhi@mail.sysu.edu.cn](mailto:wangzhi@mail.sysu.edu.cn)

*Correspondence to: Dr. Zhiyi Zuo, Department of Anesthesiology, University of Virginia Health System, 1 Hospital Drive, PO Box 800710, Charlottesville, Virginia 22908-0710. Tel: 434-924-2283, Fax: 434-924-2105. E-mail: zz3c@virginia.edu

*Correspondence to: Zhi Wang, Department of Anesthesiology, Sun Yat-sen Memorial Hospital, Sun Yat-sen University, No. 107 YanJiang West Road, Guangzhou, Guangdong Province 510289, China. E-mail: wangzhi@mail.sysu.edu.cn


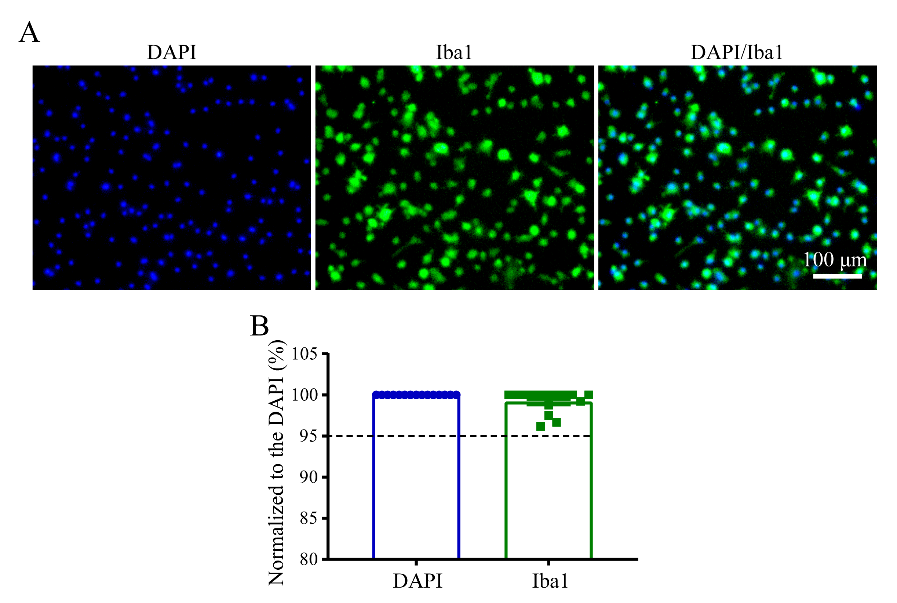


Fig. S1 The purity of cultured primary microglia was over 95%. (A) Representative images of DAPI (blue), Iba1(green) and their merge captured from cultured primary microglia. (B) Quantification of the percent of Iba1^+^ normalized to the DAPI^+^. Images were randomly taken from 15 random fields.


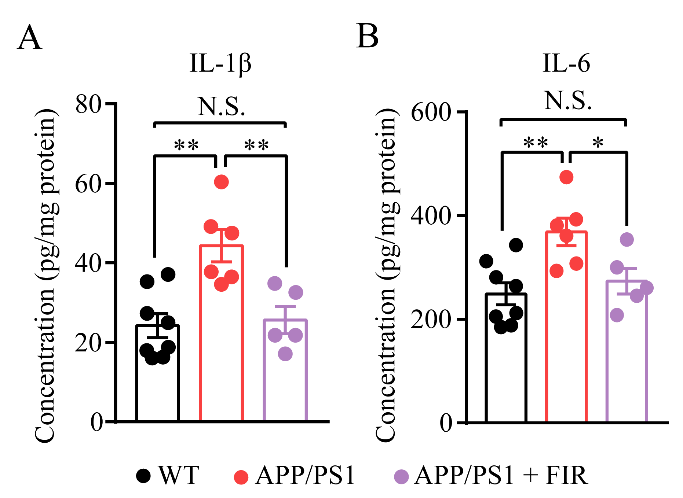


Fig. S2 FIR light alleviated neuroinflammation in the AD mice. (A) IL-1β levels and (B) IL-6 levels in the cerebral cortex. Data were means ± SEM, n = 5 - 8, *p < 0.05, **p < 0.01. N.S., not significant.


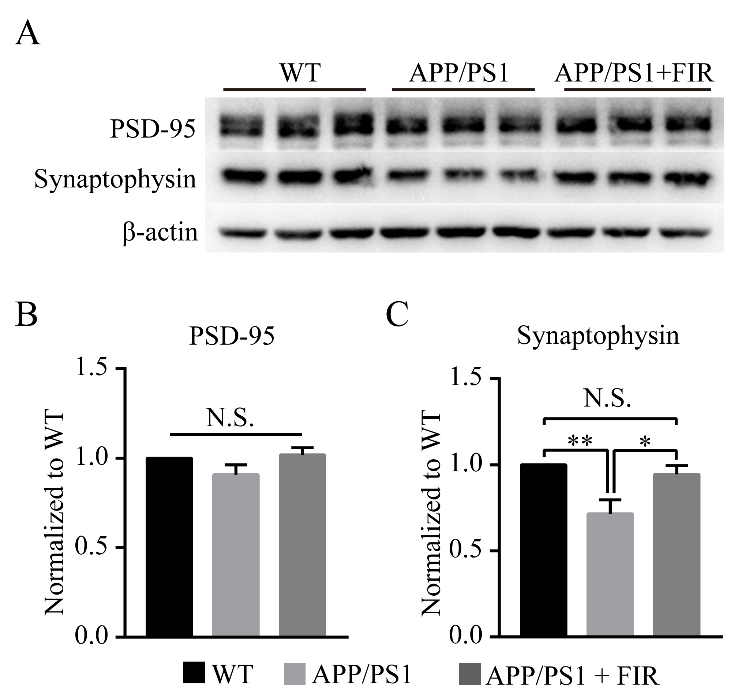


Fig. S3 FIR light restored the expression of synaptic protein. (A) The Western blot analysis of PSD-95 and synaptophysin protein in the hippocampus region of AD mice. (B) Quantification of PSD-95. (C) Quantification of synaptophysin. Data were means ± SEM, n = 5, *p < 0.05, **p < 0.01. N.S., not significant.


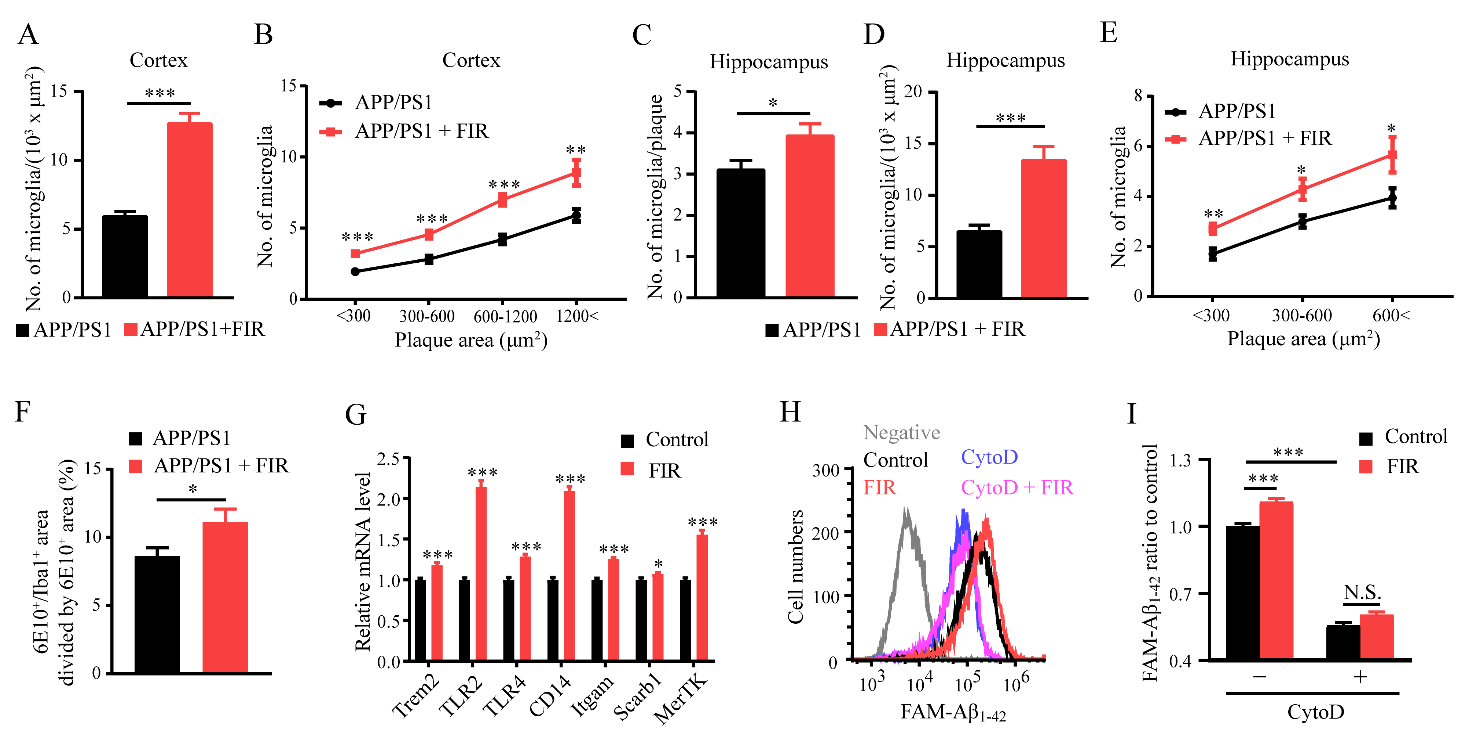


Fig. S4 FIR light enhanced microglial Aβ phagocytosis. (A) Quantification of microglial cells normalized to Aβ plaque area (10^3^ × μm^2^) in the cerebral cortex (n = 102 to 103 plaques per group). (B) Quantification of microglia per plaque of different sizes in the cerebral cortex. (C) Quantification of microglial cells within 20 μm from the Aβ plaque boundary (n = 40 plaques per group) in the hippocampus. (D) Quantification of microglial cells normalized to Aβ plaque area (10^3^ × μm^2^) in the hippocampus (E) Quantification of microglia per plaque of different sizes in the hippocampus. (F) Quantification of the percentage of 6E10^+^/Iba1^+^ co-staining area normalized to the total 6E10^+^ area (n = 49 - 52 per group). (G) Relative mRNA levels of related phagocytic receptors in primary cultured microglia (n = 6). (H) Representative histogram displaying the distribution of FAM-Aβ_1-42_-positive microglia performed by flow cytometry. (I) Quantification of the mean fluorescence of FAM-Aβ_1-42_ engulfed by microglia using flow cytometry (n = 6). Data are means ± SEM, *p < 0.05, **p < 0.01, ***p < 0.001. N.S., not significant.


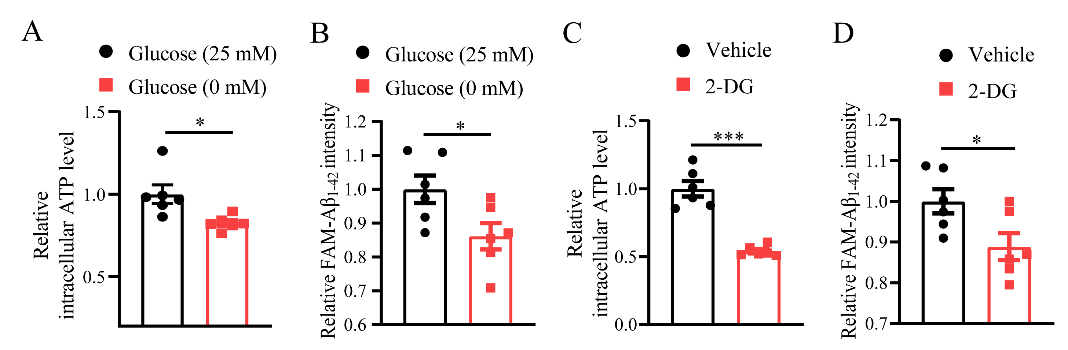


Fig. S5 Decreased intracellular ATP accompanied by compromised Aβ phagocytosis. (A) Intracellular ATP of microglia cultured in the medium containing 25 mM glucose and free glucose, respectively. (B) Aβ phagocytosis of microglia cultured in the medium containing 25 mM glucose and free glucose, respectively. (C) Intracellular ATP of microglia pretreated with 2-DG or not. (D) Aβ phagocytosis of microglia pretreated with 2-DG or not. Data were means ± SEM, n = 6, *P < 0.05, ***P < 0.001.


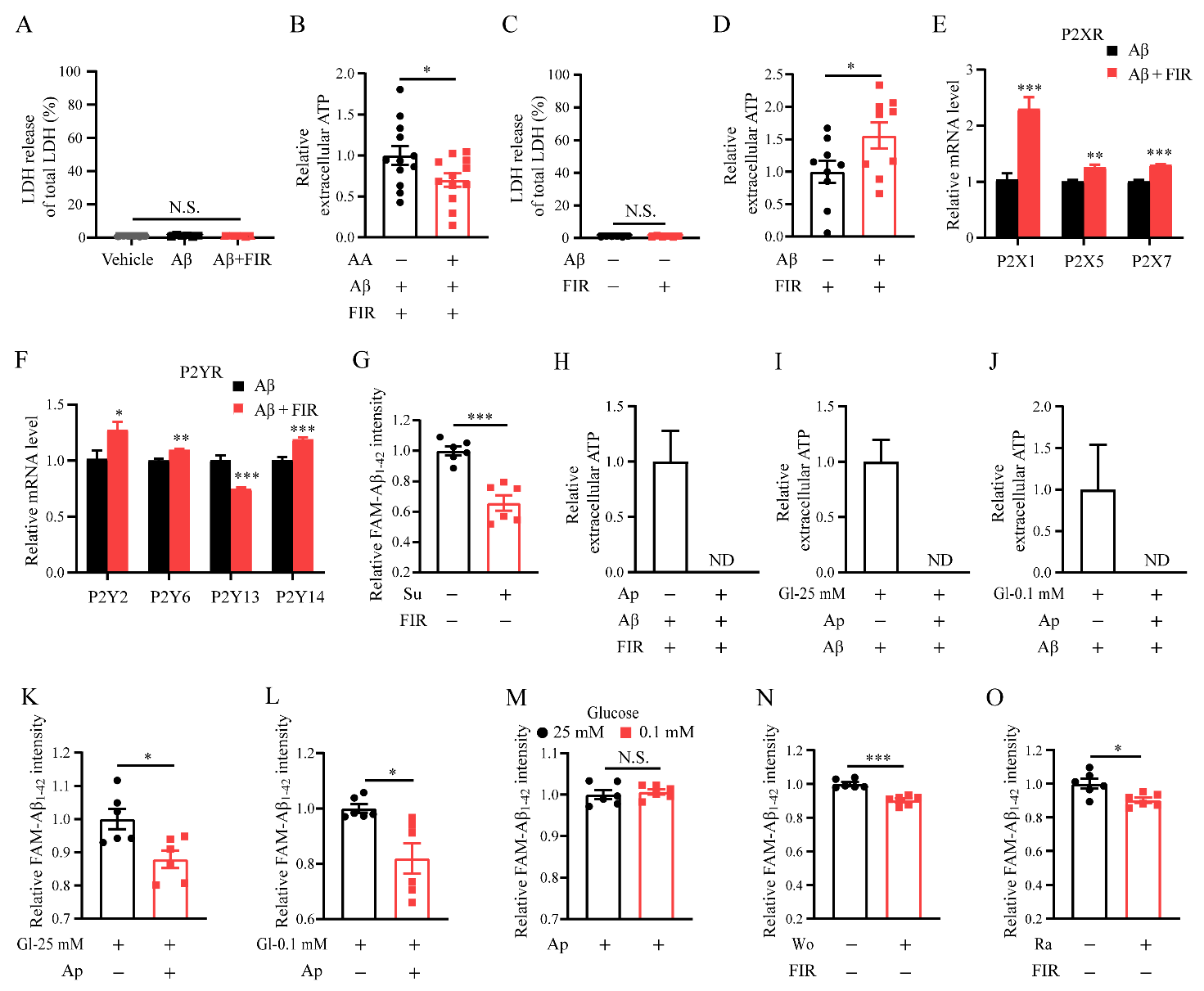


Fig. S6 FIR light enhanced microglial Aβ phagocytosis through increased microglial extracellular ATP release. (A) LDH extracellular level of microglia under different stimulation conditions (n = 6). (B) Upon the Aβ plus FIR light treatment, extracellular ATP of microglia pretreated with antimycin A or not (n = 12). (C) LDH extracellular level of microglia treated with FIR light or not (n = 6). (D) Upon the FIR light treatment, extracellular ATP of microglia treated with Aβ or not (n = 9). (E) Upon the Aβ treatment, relative mRNA level of partial ATP ligand gated cation channel receptor P2XR of microglia treated with FIR light or not (n = 6). (F) Upon the Aβ treatment, relative mRNA level of partial ATP G protein coupled receptor P2YR of microglia treated with FIR light or not (n = 6). (G) Aβ phagocytosis of microglia pretreated with suramin or not (n = 6). (H) Upon the FIR light plus Aβ treatment, extracellular ATP of microglia pretreated with apyrase or not (n = 6). (I) Pretreatment with Apyrase or not, extracellular ATP of microglia cultured in the medium containing Aβ and glucose of 25 mM (n = 6). (J) Pretreatment with Apyrase or not, extracellular ATP of microglia cultured in the medium containing Aβ and glucose of 0.1 mM (n = 6). (K) Pretreatment with apyrase or not, Aβ phagocytosis of microglia cultured in the medium containing glucose of 25 mM (n = 6). (L) Pretreatment with apyrase or not, Aβ phagocytosis of microglia cultured in the medium containing glucose of 0.1 mM (n = 6). (M) Upon the apyrase pretreatment, Aβ phagocytosis of microglia cultured in the medium containing glucose of 25 mM and 0.1 mM, respectively (n = 6). (N) Aβ phagocytosis of microglia pretreated with wortmannin or not (n = 6). (O) Aβ phagocytosis of microglia pretreated with rapamycin or not (n = 6). Data were means ± SEM, *p < 0.05, **p < 0.01, *** p < 0.001. N.S., not significant. ND, not detected. AA, antimycin A. Su, suramin. Ap, apyrase. Wo, wortmannin. Ra, rapamycin.


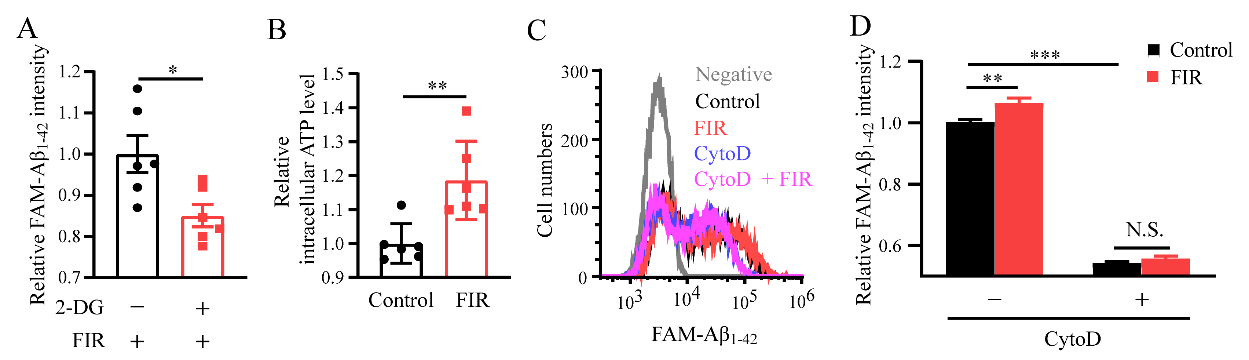


Fig. S7 FIR promoted ATP production and enhanced Aβ phagocytosis. (A) Upon the FIR light treatment, Aβ phagocytosis of microglia pretreated with 2-DG or not. (B) Intracellular ATP of BV2 microglia cells treated with FIR light or not. (C) Representative histogram displaying the distribution of FAM-Aβ_1-42_-positive BV2 microglia performed by flow cytometry. (D) Quantification of the mean fluorescence of FAM-Aβ_1-42_ engulfed by BV2 microglia using flow cytometry. Data were means ± SEM, n = 6, *p < 0.05, ** p < 0.01, *** p < 0.001. N.S., not significant. CytoD, cytochalasin D.

Supplementary Table:

Table S1. List of qPCR primers.

| Gene | Forward | Reverse |
| --- | --- | --- |
| Trem2 | CTGGAACCGTCACCATCACTC | CGAAACTCGATGACTCCTCGG |
| TLR2 | TCTAAAGTCGATCCGCGACAT | CTACGGGCAGTGGTGAAAACT |
| TLR4 | GCCTTTCAGGGAATTAAGCTCC | GATCAACCGATGGACGTGTAAA |
| CD14 | CTCTGTCCTTAAAGCGGCTTAC | GTTGCGGAGGTTCAAGATGTT |
| Itgam | CCATGACCTTCCAAGAGAATGC | ACCGGCTTGTGCTGTAGTC |
| Scarb1 | CGAAGTGGTCAACCCAAACGA | CCATGCGACTTGTCAGGCT |
| MerTK | CCTAACCGTACCTGGTCTGAC | GGGAGGGGATTACTTTGATGTTG |
| P2X1 | CGGATGGTGCTGGTACGAAA | CACTGACACACTGCTGATAAGG |
| P2X5 | ACCAACCTGATCGTGACTCCT | ACAGTCGGTGTCCTCTGAACA |
| P2X7 | CAGCGGAAAGAGCCTGTTATC | TGGCCTTCTGACTTGACATAGTT |
| P2Y2 | TGTTGCCCGTGTCCTATGG | GCGTAGAGAGAGTCCGAAACTG |
| P2Y6 | GACCTGATGTATGCCTGTTCAC | CAGGATGCTGCCATGTAGATTG |
| P2Y13 | ATGCTCGGGACAATCAACACC | CCACAGTATAGAGAACCGGGA |
| P2Y14 | TGGCACAAGGCGTCTAACTAT | GACTTCCTCTTGACGGAGGTG |
| β-actin | GTGACGTTGACATCCGTAAAGA | GCCGGACTCATCGTACTCC |
